# Mercury Induced Tissue Damage, Redox Metabolism, Ion Transport, Apoptosis, and Intestinal Microbiota Change in Red Swamp Crayfish (*Procambarus clarkii*): Application of Multi-Omics Analysis in Risk Assessment of Hg

**DOI:** 10.3390/antiox11101944

**Published:** 2022-09-29

**Authors:** Lang Zhang, Yuntao Zhou, Ziwei Song, Hongwei Liang, Shan Zhong, Yali Yu, Ting Liu, Hang Sha, Li He, Jinhua Gan

**Affiliations:** 1 Yangtze River Fisheries Research Institute, Chinese Academy of Fishery Sciences, Wuhan 430223, China; 2 Department of Genetics, Wuhan University, Wuhan 430071, China; 3 Hubei Province Key Laboratory of Allergy and Immunology, Wuhan 430071, China; 4 Key Laboratory of Control of Quality and Safety for Aquatic Products, Ministry of Agriculture and Rural Affairs, Beijing 100141, China

**Keywords:** *Procambarus clarkii*, mercury, histopathology, intestinal microbiota, hepatopancreatic transcriptome

## Abstract

As one of the most toxic elements, mercury (Hg) is a widespread toxicant in aquatic environments. Crayfish are considered suitable for indicating the impact of heavy metals on aquatic crustaceans. Nevertheless, Hg toxicity on *Procambarus clarkii* is largely unknown. In this research, the acute Hg-induced alterations of biochemical responses, histopathology, hepatopancreatic transcriptome, and intestinal microbiome of *Procambarus clarkii* were studied. Firstly, Hg induced significant changes in reactive oxygen species (ROS) and malonaldehyde (MDA) content as well as antioxidant enzyme activity. Secondly, Hg exposure caused structural damage to the hepatopancreas (e.g., vacuolization of the epithelium and dilatation of the lumen) as well as to the intestines (e.g., dysregulation of lamina epithelialises and extension of lamina proprias). Thirdly, after treatment with three different concentrations of Hg, RNA-seq assays of the hepatopancreas revealed a large number of differentially expressed genes (DEGs) linked to a specific function. Among the DEGs, a lot of redox metabolism- (e.g., ACOX3, SMOX, GPX3, GLO1, and P4HA1), ion transport- (e.g., MICU3, MCTP, PYX, STEAP3, and SLC30A2), drug metabolism- (e.g., HSP70, HSP90A, CYP2L1, and CYP9E2), immune response- (e.g., SMAD4, HDAC1, and DUOX), and apoptosis-related genes (e.g., CTSL, CASP7, and BIRC2) were identified, which suggests that Hg exposure may perturb the redox equilibrium, disrupt the ion homeostasis, weaken immune response and ability, and cause apoptosis. Fourthly, bacterial 16S rRNA gene sequencing showed that Hg exposure decreased bacterial diversity and dysregulated intestinal microbiome composition. At the phylum level, there was a marked decrease in *Proteobacteria* and an increase in *Firmicutes* after exposure to high levels of Hg. With regards to genus, abundances of *Bacteroides*, *Dysgonomonas*, and *Arcobacter* were markedly dysregulated after Hg exposures. Our findings elucidate the mechanisms involved in Hg-mediated toxicity in aquatic crustaceans at the tissue, cellular, molecular as well as microbial levels.

## 1. Introduction

In recent years, there has been increasing concern about aquatic heavy metal pollution [1,2,3]. Mercury, a toxic element, reaches aquatic environments mainly via anthropogenic activities. Globally, it is the third most common environmental contaminant [4,5]. Mercury is transported and biomagnified in aquatic ecosystems via aquatic animal food webs, such as algae, sediments, carnivorous fish, and benthic crustaceans. Methyl mercury (MeHg), a Hg compound, is obtained from Hg^2+^ via microbial activities [6]. It bio-accumulates in higher trophic consumers, particularly long-living, slow-growing species [7,8,9]. In lakes as well as estuaries, sediments are common sites for MeHg [10,11,12,13]. However, anthropogenic mercury input is highly associated with elevated Hg^2+^ levels. Thus, the accumulation of Hg^2+^ in benthic crustaceans found at the sediment and water interface is a focus of research [14,15]. Unfortunately, in crustaceans, acute toxic responses to inorganic Hg have not been fully established.

Adverse outcomes of Hg on organisms have been documented. Toxic effects of mercury on animal models (e.g., fish and mouse), depending on their exposure duration and dose, could cause hepatotoxicity [16], neurotoxicity [17], as well as endocrine [18] and reproductive disruption [19]. The toxicity of Hg on crustaceans has also been documented; however, most of them were focused on the larval stage and the median lethal concentration (LC50). For instance, 96 h Hg LC50 values in various species were: 20 μg/L in *Penaeus monodon* postlarvae [20], 1.2 μg/L in *Penaeus japonicus* postlarvae [21], 15 μg/L in *Neomysis awatschensis* [22], 18 μg/L in *Penaeus japonicus* embryos [23], and 40 μg/L in *Scylla serrata* juveniles [24]. Despite these studies, more research should be undertaken to determine the impact of Hg on antioxidant enzymes, histopathology, hepatopancreatic transcriptome, and intestinal microbiome structures of crustaceans.

It has been found that metals induced the production of ROS, which causes oxidative stress, resulting in several detrimental effects on cells [25]. The overproduction of ROS leads to the formation of malonaldehyde-like species in lipids [26,27]. Consequently, ROS and MDA levels reflect the degree of oxidative damage. In order to protect themselves from ROS, organisms (such as crustaceans) have mechanisms of non-enzymatic and enzymatic antioxidants [28]. Non-enzymatic antioxidants contain tocopherols, ascorbic acid, and glutathione (GSH). GSH is the most abundant cellular thiol and well-studied antioxidant compound in organisms [29]. In terms of enzymatic defenses, superoxide dismutase (SOD), catalase (CAT), and glutathione S-transferase (GST) are antioxidant enzymes responsible for maintaining cellular redox status [30]. Higher enzyme activity means higher detoxification capacity, which is important for counteracting ROS-induced cellular damage [31,32]. However, not much is known concerning the effects of Hg on oxidative stress damage and the antioxidant system in crustaceans.

In crustaceans, the hepatopancreas is involved in metabolism, immune functions, nutrient absorption, and xenobiotic detoxification [33,34]. In addition, the hepatopancreas is one of the key organs affected by environmental stressors [35,36,37]. In crustaceans, transcriptome analysis is an effective method to provide information about the global expression profiles of genes and related mechanisms involved in the toxicity of heavy metals [38,39,40]. The intestinal microbiome is important in the sustenance of health and in the regulation of many vital physiological host functions [41,42,43]. Studies on gut microbial communities suggest that diseased and healthy shrimp have different intestinal bacterial communities [43]. In addition, gut microbiota can be used to measure shrimp health [44]. Research on crustaceans has revealed the importance of diets [45], developmental stage [46], health status [43], and risk factors [47,48] on the gut microbiota. Furthermore, the relationship between heavy metal toxicity and intestinal microbiota alteration in crustaceans has been studied. The concentration of 0.5 mg L^−1^ Cu^2+^ or more increases the abundance of intestinal pathogens in *Litopenaeus vannamei* [49]. Cd exposure could alter the richness, diversity, and composition of intestinal microbiota in *Procambarus clarkii* (*P. clarkii*) [47]. However, the toxicity of inorganic Hg to crustaceans has not been fully established. Deep sequencing data of hepatopancreatic transcriptome and intestinal microbiota will reveal abundant genetic and bacterial signatures of Hg toxicity.

*Procambarus clarkii*, the freshwater crayfish is an important commercial species [50,51]. Considering its long lifecycle, wide distribution, and simple anatomy, *P. clarkii* is often utilized as a typical bioindicator of toxic pollutants in studies on aquatic environments [38,52,53,54]. Additionally, *P. clarkii* is considered a model organism for research in aquatic crustaceans [55]. In the present research, adult *P. clarkii* was exposed to acute Hg. On the basis of these previous studies, we investigated the bioaccumulation together with antioxidant enzymes, histological variations, the hepatopancreatic transcriptome, and intestinal microbiota changes to further reveal how *P. clarkii* responds to inorganic Hg^2+^ at the biochemical, physiological, molecular, and intestinal microbiota levels. Another crucial question in our study is how Hg exposure damages crustaceans’ tissues. The results of this study could serve as a physiological reference for mitigating the negative effects of Hg stress on crustacean aquaculture.

## 2. Materials and Methods

### 2.1. Experimental Animals

To avoid gender-related differences and the influence of female oviposition, only male adult freshwater crayfish with similar sizes (weight: 19.46 ± 2.84 g, length: 9.8 ± 1.4 cm) were acquired from a commercial crayfish farm in Lixian (Changde, China). They were kept in several glass aquaria (60 cm length, 40 cm width, and 35 cm depth) containing 30 L of de-chlorinated tap water (temperature 24.0 ± 0.8 °C, CaCO_3_ hardness 44.21 ± 0.28 mg/L, pH 7.10 ± 0.07, and DO 6.76 ± 0.21 mg/L) for 7 days for acclimatization. Before experimental procedures, all crayfish were fed once daily on red worms (*Limnodrilus*), but the crayfish were not fed during exposure experiment. In addition, the photoperiod was 12 h/12 h dark/light.

### 2.2. Toxicity Test

**Figure 1:**
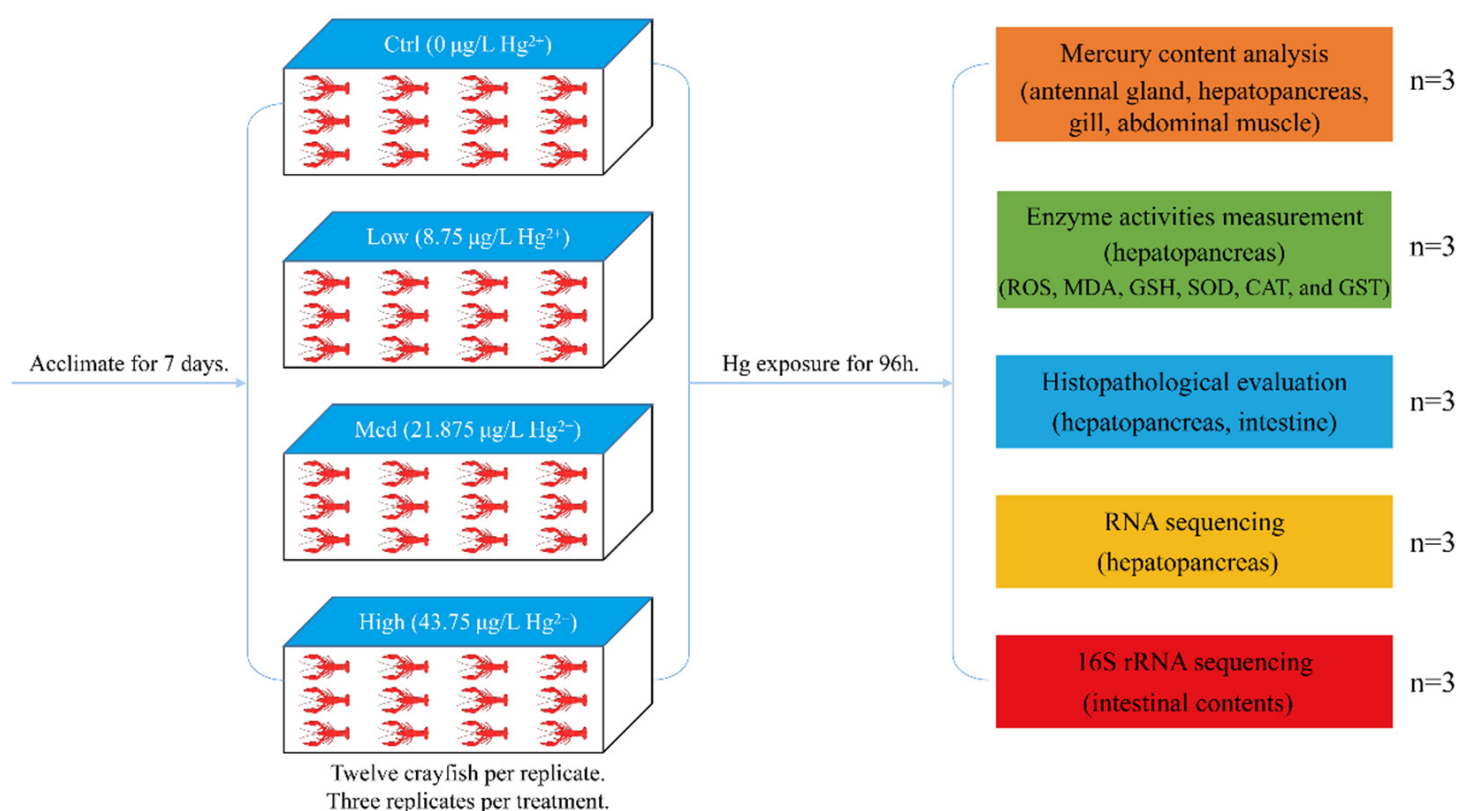
Schematic representation of the experimental procedure.

Crayfish were randomized into 4 groups (three replicates per group); in addition, twelve specimens per replicate. There was no replacement of water during the static exposure experiment. HgCl_2_ (Sinopharm Chemical Reagent Company, Shanghai, China) at analytical grades was used. Previous study has indicated that 96 h LC50 concentration of Hg^2+^ for freshwater crayfish at 24 °C is 0.35 mg/L [56], in order to better evaluate the relationship between Hg concentration and toxicity; therefore, an appropriate Hg^2+^ exposure time of 96 h and temperature of 24 °C were chosen for this toxicity test. The Hg exposure concentration drew on research conducted with another heavy metal cadmium exposure on the crayfish [38], exposures of *P. clarkii* to Hg were respectively conducted at doses of 0 (Control group, Ctrl, 0 μg/L Hg^2+^), 1/40 LC50 (Low concentration group, Low, 8.75 μg/L Hg^2+^), 1/16 LC50 (Medium concentration group, Med, 21.875 μg/L Hg^2+^), and 1/8 LC50 (High concentration group, High, 43.75 μg/L Hg^2+^). Figure 1 illustrates the experimental procedure schematically. The doses of Hg^2+^ solutions were established by dissolving the desired amount of Hg^2+^ stock solution in dechlorinated tap water. The other exposure conditions were similar to those for the acclimatization described above. There was no crayfish death during the exposure experiments.

### 2.3. Sampling

In the Hg^2+^ exposure experiment, all samples from each group were randomly selected at 96 h and anesthetized with eugenol bath (1:10,000). To examine histopathology, crayfish hepatopancreas and gut tissue samples were fixed in 4% paraformaldehyde. To avoid inter-individual variation, tissues of three specimens were pooled together per biological replicate. Three replicates of each Hg^2+^ treatment were collected for the analysis of total mercury content, enzyme activities, hepatopancreas transcriptome, as well as intestinal microbiota. To assess transcriptomic changes caused by Hg^2+^, 300 mg of the hepatopancreas were acquired, instantly frozen in liquid nitrogen, and then kept at −80 °C for RNA extractions. Subsequently, intestines were flushed thrice using PBS and dissected. Then, intestinal contents were cautiously obtained in a 1.5 mL sterile centrifuge tube, immediately frozen in liquid nitrogen, and kept at −80 °C until required. During sampling, all operations were conducted on super clean workbench.

### 2.4. Total Mercury Content Analysis

At 0 h and 96 h, water samples (10 mL) from each aquarium were taken and acidified with HNO_3_ for later use. Samples of crayfish were digested according to a previous publication [57]. In order to digest the tissue samples, a microwave digestion system (MARS, CEM) was utilized. A total of 300 mg of each sample and 10 mL mixed liquid of HClO_4_ (perchloric acid 70%) and HNO_3_ (Nitric acid 65% Suprapur^®^) were added to the digestion vessel. Following is the digested program: 5 min to temperature 120 °C, 5 min at temperature 120 °C, 5 min to temperature 150 °C, 10 min at temperature 150 °C, 5 min to temperature 190 °C, and 20 min at temperature 190 °C. Once the cooling process is complete, 2% HNO3 was added to the digestion solution to dilute it to 50 mL for later use. The concentration of Hg was determined by atomic fluorescence spectrometer (AFS, Wuhan, China).

### 2.5. Measurement of Enzyme Activities

The protein concentration, content of ROS (Cat. No. E004-1-1), MDA (Cat. No. A003-1-1) as well as GSH (Cat. No. A006-2-1), and enzyme activities of SOD (Cat. No. A001-3-2), CAT (Cat. No. A007-1), as well as GST (Cat. No. A004-1-1), were examined using Testing Kit (Nanjing Jiancheng Bioengineering, Nanjing, China). DCFH oxidation method by Keston and Brandt was used to determine the ROS levels in hepatopancreas [58]. SOD was quantified based on the method of Marklund and Marklund [59]. MDA, CAT, and GST were measured based on the methods of Satoh [60], Sinha [61], and Habig et al. [62], respectively. Concentrations of GSH were estimated by the method of Moron et al. [63]. The levels of ROS, MDA, GSH, SOD, CAT, and GST were normalized with the corresponding protein content.

### 2.6. Histopathological Evaluation

Dehydration of the fixed hepatopancreas as well as the gut was performed through a graded-ethanol serial, made transparent by soaking in xylene, and then paraffin-embedded. After that, sections (4 μm thick) were prepared utilizing a rotary microtome followed by hematoxylin and eosin staining. At last, stained sections were analyzed by a microscope (Olympus IX73).

### 2.7. RNA Isolation, Preparation of the RNA-Seq Library, and Sequencing

Isolation of total RNA from the hepatopancreas was performed using the Tri Reagent, as instructed by the manufacturer. With the Agilent Bioanalyzer 2100 system (Agilent Technologies, Shanghai, China), RNA Nano 6000 Assay Kit was used for the evaluation of RNA integrity and quantification. In accordance with the manufacturer’s instructions, sequencing libraries were prepared using NEBNext^®^ Ultra^TM^ RNA Library Prep Kit for Illumina^®^ (NEB, Ipswich, MA, USA). After DNase I treatment, poly-T oligo (dT) magnetic beads were used to purify mRNA from RNA. After purification and fragmentation, the mRNA was utilized to synthesize cDNA. The downstream experiments were conducted on samples with an RNA integrity number (RIN) > 7.0. SMARTer PCR cDNA synthesis kit was used to prepare libraries for RNA sequencing. Twelve cDNA libraries were constructed from the Ctrl, Low, Med, and High groups, each group with three biological replications. To generate paired-end reads from the libraries, the Illumina HiSeq 2500 sequencing platform was used.

### 2.8. Transcriptome Assembly and Annotation

The in-house Perl scripts were initially used to process raw reads in fastq format. To obtain clean reads, adaptor sequences, low-quality sequences, as well as poly-N were eliminated from raw reads. Subsequently, Q20, Q30, and GC levels of the clean reads were evaluated. The Raw RNA-seq data were deposited in the NCBI Sequence Read Archive (SRA) under BioProject PRJNA788175 (www.ncbi.nlm.nih.gov/bioproject/PRJNA788175, accessed on 12 December 2021). Additionally, de-novo assembly of *P. clarkii* transcriptome was accomplished utilizing Trinity software [64]. Gene function annotation was based on GO (gene ontology), KO (KEGG Ortholog), NR, Pfam (protein family), STRING, SWISSPROT, and KOG databases.

### 2.9. Identification of DEGs

DEGs were identified by “DESeq” in R [65]. Adjustments of *p* values were made to control the FDR (false discovery rate) [66]. Genes with log_2_ |Fold Change| > 1 and adjusted *p*-value (FDR) < 0.05 were assigned as DEGs. The R function prcomp was used to perform the principal component analyses (PCA) for all genes. Hierarchical DEG clustering was conducted using the R ‘heatmap’ package. Then, WEGO as well as Blast2GO v2.5 programs were used to map the DEGs to the GO database for functional annotations. Furthermore, all DEGs were mapped to terms in the KEGG database to establish the markedly enriched KEGG terms.

### 2.10. Quantitative RT-PCR (qPCR) Assay

Primers of peroxisome-related genes were designed utilizing Primer Premier 6 software. 18S rRNA gene was utilized as the reference “housekeeping” gene of *P. clarkii* according to previous publications [38,51]. The primer sequences, amplicon size, and amplification efficiency were displayed in Table S1. According to the documentation, qPCR was performed using SYBR Green [67,68]. Briefly, qPCR was performed in 20 μL reactions comprised of 2 μL of cDNA, 0.4 μL of 10 μM both the reverse as well as forward primers, 10 μL of 2 × SybrGreen qPCR Master Mix, as well as 7.2 μL of RNase-free H_2_O. The PCR thermal cycle program consisted of 95 °C for 2 min, 45 cycles of 95 °C for 3 s, and 60 °C for 30 s. Analysis of relative expressions was conducted using the 2^−^^△△Ct^ method [69].

### 2.11. Extraction of DNA and PCR-Amplifications

Total intestinal DNA was extracted using the E.Z.N.A.^TM^ Mag-Bind Soil DNA Kit (Omega Bio-Tek, Norcross, GA, USA). PCR amplification of the 16S rDNA hypervariable V3-V4 regions was performed using primers 338F 5′-ACTCCTACGGGAGGCAGCA-3′ and 806R 5′-GGACTACHVGGGTWTCTAAT-3′. All PCR reactions were conducted in triplicate using a total volume of 20 μL reaction system containing 2 μL 2.5 mM dNTPs, 4 μL 5 × FastPfu Buffer, 0.4 μL FastPfu Polymerase, 0.8 μL each primer (5 mM), and 10 ng template DNA. According to Zhang et al., PCR amplification was conducted [37].

### 2.12. Illumina Miseq and Sequencing

The AxyPrep DNA Gel Extraction Kit (Axygen Biosciences, Union City, CA, USA) was used to extract amplicons from 2% agarose gels after which they were purified. Quantification of the PCR product was performed using the QuantiFluor^TM^-ST fluorescence system (Promega, Madison, WI, USA). After that, the purified amplicons were pooled in equimolar concentrations, then sequenced (2 × 300) on an Illumina MiSeq platform. Demultiplexed 16S rRNA data were quality-filtered using the Quantitative Insights Into Microbial Ecology (QIIME 1.8.0) software package. Firstly, low-quality reads with scores < 20 or with a read length < 200 bp were filtered out. Secondly, barcodes were matched, while ambiguous bases and unmatched barcodes were removed. Thirdly, unassembled reads were discarded. Overlapping sequences longer than 10 bp were assembled based on their overlap sequences. UPARSE 7.1 (http://drive5.com/uparse/ accessed on 12 December 2021) was used to cluster operational taxonomic units (OTUs). UCHIME was used to identify and remove chimeric sequences. Using a confidence threshold of 70%, RDP Classifier (http://rdp.cme.msu.edu/ accessed on 12 December 2021) was used to analyze the taxonomy of individual 16S rRNA gene sequences against Silva (SSU115). Sequences obtained by 16S rRNA sequencing were deposited in NCBI SRA under BioProject PRJNA788294 (www.ncbi.nlm.nih.gov/bioproject/PRJNA788294, accessed on 12 December 2021).

### 2.13. Biodiversity Analysis

Alpha diversity assessments, such as Community coverage index (Coverage), diversity parameters (Simpson, Shannon), and richness parameters (Ace, Chao) were performed using the MOTHUR software (v.1.30.1) [70]. QIIME (version 1.8.0) was used to construct the rarefaction curves to evaluate sequencing depth. As for measurements of beta diversity, unweighted Unifrac was utilized for Principal Coordinate Analysis (PCoA). Unweighted pair-group method with arithmetic means (UPGMA) hierarchical clustering was performed using QIIME and displayed using R. Venn diagrams were performed using R to display shared, unique OTUs (operational taxonomic units) [71]. Diagrams of intestine microbial community composition were plotted utilizing Origin 8.0 software. Differences in communal composition differences between the Hg^2+^ exposure and control groups were evaluated by using one-way ANOVA, with *p* ≤ 0.05 signifying statistical significance.

## 3. Results

### 3.1. The Bioaccumulation of Hg in the Tissues of P. clarkii

As shown in Table S2, a slight decrease in Hg concentration in water was observed over time due to accumulation by crayfish and adsorption on aquarium walls. After exposure for 96 h, the measured Hg concentrations (mean ± SD) were 0, 8.56 ± 0.16, 21.39 ± 0.42, and 43.72 ± 0.84 μg/L in Control, Low, Med, and High group, respectively.

Bioaccumulation of Hg in tissues during Hg^2+^ exposure is shown in Table S3. The crayfish in control group showed very low Hg accumulation levels. In control tissues, Hg accumulation levels were relative to each other as follows: gill > hepatopancreas > abdominal muscle > antennal gland. With increasing Hg concentrations, the accumulation level of Hg in all examined tissues significantly increased (*p* < 0.05) after 96 h of exposure. In all tissues with Hg^2+^-treated for 96 h, Hg accumulation levels were relative to each other as follows: gill > antennal gland > hepatopancreas > abdominal muscle. These results indicated that Hg bioaccumulation in tissues (gill, antennal gland, hepatopancreas, abdominal muscle) was dose-dependent.

### 3.2. Oxidative Stress and Antioxidant Parameters

As a result of Hg exposure, enzymes involved in the response to oxidative stress were affected. As shown in Table S4, the ROS and MDA content of hepatopancreas in all Hg-treated crayfish increased significantly (*p* < 0.05). After Hg exposure for 96 h, ROS levels in Low, Med, and High groups increased by 37.21%, 41.24%, and 57.20%, respectively. MDA levels increased by 23.77%, 29.60%, and 37.22%, respectively. With increasing Hg concentrations, the enzymatic activities of hepatopancreatic SOD and CAT decreased gradually. The effects of Hg exposure on glutathione-mediated antioxidant enzyme activities were also significant. Concentrations of GSH in crayfish hepatopancreas were 17.45%, 23.73%, and 26.86% less, respectively, relative to that of control. Activity of GST in crayfish hepatopancreas was 14.48%, 19.26%, and 26.46% greater, respectively, relative to that of control.

### 3.3. Histopathology

**Figure 2:**
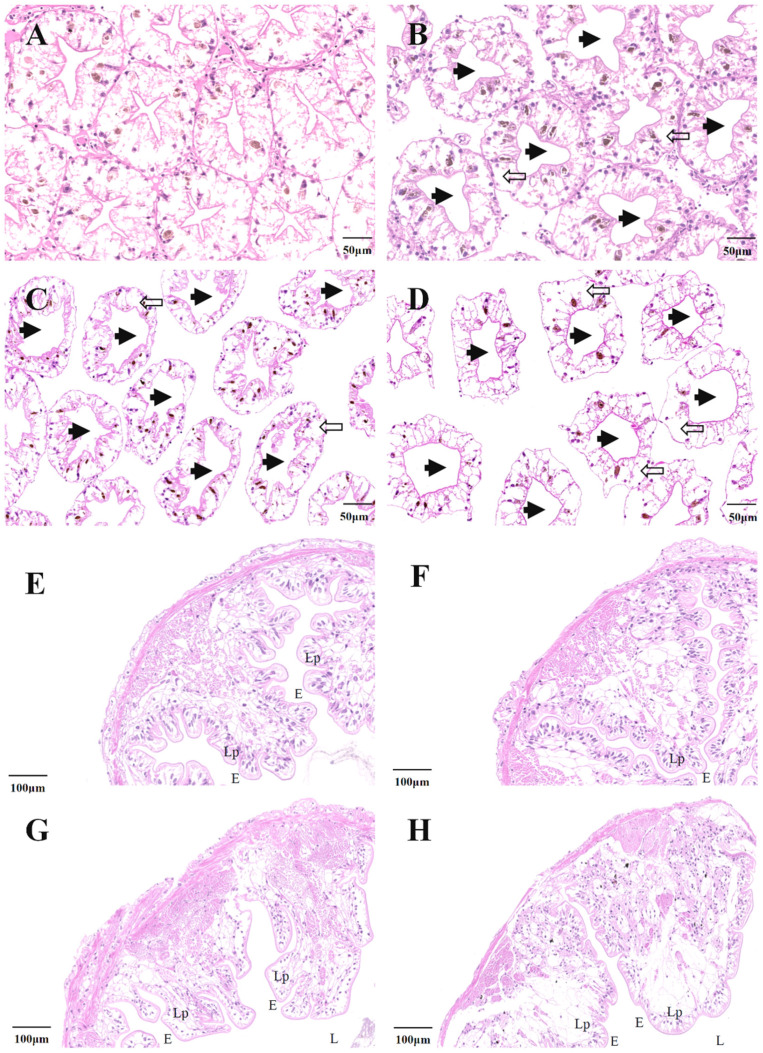
Hg exposure caused hepatopancreas and intestines injury. Hepatopancreas histology from Ctrl (**A**), Low (**B**), Med (**C**), and High (**D**) group in 96 h. Intestines histology from Ctrl (**E**), Low (**F**), Med (**G**), and High (**H**) group in 96 h. Black solid arrow: dilatation of tubule lumen; Black hollow arrow: vacuolization; Lp: lamina propria; E: epithelium; L: lumen. H&E stain (100×). Ctrl: 0 μg/L Hg^2+^; Low: 8.75 μg/L Hg^2+^; Med: 21.875 μg/L Hg^2+^; High: 43.75 μg/L Hg^2+^.

Histological sections from hepatopancreas and intestines of Ctrl and Hg^2+^-treated crayfish are shown in Figure 2. As shown in Figure 2A, the hepatopancreas cells from Ctrl group displayed well-organized structures and asterisk-like shapes in the tubule lumens. As shown in Figure 2B–D, compared with Ctrl group, the hepatopancreas cells of crayfish treated with Hg exhibited histological changes. All Hg-treated (Low, Med, and High concentrations of Hg^2+^) hepatopancreas cells exhibited tubule lumen dilatations and apparent epithelium vacuolizations. In addition, this research demonstrated that crayfish intestines treated with Hg^2+^ displayed signs of damage. The intestine cells from Ctrl group showed normal palisade arrangements and a regular nucleus (Figure 2E). Compared with Ctrl group, the intestine in Low group was nearly identical (Figure 2F). By contrast, the crayfish intestines treated with Med and High concentrations of Hg displayed histological differences from intestines of Ctrl group, including apparent vacuoles in the microvilli, extended lamina proprias, and disordered lamina epithelialises (Figure 2G–H). As shown in Figure S1, increased Hg concentration significantly increased the proportions of hepatopancreatic tubule lumen dilatation and intestine microvilli vacuolization. After Hg exposure for 96 h, the mean proportion of hepatopancreatic tubule lumen dilatation in Control, Low, Med, and High groups were 5.6%, 32.9%, 63.8%, and 84.7%, respectively. The mean proportion of intestine microvilli vacuolization in Control, Low, Med, and High groups were 5.4%, 9.9%, 33.8%, and 44.7%, respectively. The above results indicate that the degree of hepatopancreas and intestine tissue damage showed a dose-dependent relationship with the concentration of Hg.

### 3.4. Transcriptome Sequencing and Assembly

In this study, we constructed cDNA libraries of mRNAs using the hepatopancreas RNA isolated from the Ctrl and Hg-treated groups. RNA-seq generated 42,795,604 to 61,860,116 raw reads (Table S5). Subsequent to the initial quality control, 42,694,670 to 61,735,916 clean reads (99.69–99.82% of the raw data) were generated. From the 12 constructed libraries, the clean reads were utilized for sequence assembly. Additionally, a total of 192,666 unigenes were generated by de novo assembly of RNA sequencing data using Trinity software. The length distribution of the unigenes was as presented in Figure S2. The average, largest, and smallest lengths for all unigenes were 711.6 bp, 36,069 bp, and 197 bp, respectively. 

### 3.5. Functional Annotations and Classification

In our study, after transcriptome assembly, A total of 192,666 unigenes were identified from the hepatopancreas of *P. clarkii*. By using BLASTx and BLASTn, 192,666 unigenes were further classified based on their functional predictions. BLAST results showed that 6006, 5091, 11564, 5357, 7694, 6068, and 6409 unigenes matched with the annotated sequences in GO, KO, NR, PFAM, STRING, SWISSPROT, and KOG databases, respectively (Table S6). 

GO classification is a standardized system to categorize genes. According to Figure S3A–C, 31, 20, and 15 of these subcategories were grouped into biological processes, cellular components, and molecular functions, respectively. Among the biological processes, cellular processes were the most abundant with 5478 unigenes, metabolic processes with 4828 unigenes, and biological regulation with 4293 unigenes. Among the sequences categorized as molecular function, 4924 unigenes were included in binding and 3245 unigenes were predicted to possess catalytic activity. Among the sequences categorized as component categories, cell was the most abundant with 5535 unigenes, cell part with 5530 unigenes, and organelle with 5178 unigenes, which are involved in the basic functional and structural unit of organisms. These results indicated that most of the annotated unigenes were related to various types of biological processes. KOG database classified 6409 unigenes into 25 functional categories (Figure S3D). The predominant category contains signal transduction mechanisms, general function prediction only, cytoskeleton, function unknown, ribosomal structure and biogenesis, transcription, and translation. In the KEGG pathway database, based on the KO database, a total of 5091 unigenes were grouped into 364 pathways. The majority of the unigenes were divided into the categories of signal transduction (813), transport and catabolism (502), endocrine system (466), immune system (404), and translation (394). The top 34 of these KEGG biological pathway classifications are shown in Figure S3E.

### 3.6. The DEGs

**Figure 3:**
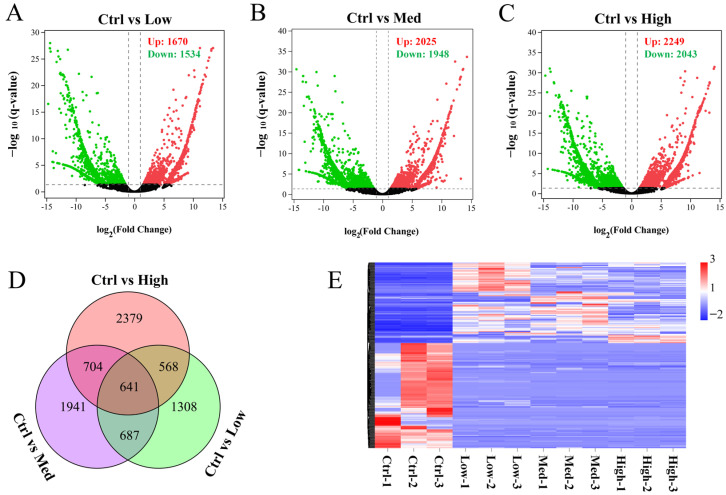
DEGs were identified after treatment with different concentrations of Hg. Volcano plots for DEGs in the 3 comparisons: (**A**) Ctrl vs. Low; (**B**) Ctrl vs. Med; (**C**) Ctrl vs. High. The red dots indicate genes that are up-regulated, while the green dots indicate genes that are down-regulated. (**D**) Venn diagram of DEGs in the 3 comparisons. (**E**) Heatmap based on fragments per kilobase of transcript per million mapped reads (FPKM) values showing the variations in expressions of overlapping DEGs. Genes whose expressions were greater than the mean are colored red while those below the mean are colored blue. Ctrl: 0 μg/L Hg^2+^; Low: 8.75 μg/L Hg^2+^; Med: 21.875 μg/L Hg^2+^; High: 43.75 μg/L Hg^2+^.

Biological repetition is particularly important for biological experiments. To do so, principal component analysis was used, as shown in Figure S4; the 3 parallels in each group are very similar, indicating general comparability between the four groups. To evaluate Hg toxicity, DEGs in Low, Med, and High groups relative to Ctrl group hepatopancreas were determined. Compared with Ctrl group, 1670 genes were up-regulated, and 1534 genes were down-regulated in Low group; 2025 genes were up-regulated, and 1948 genes were down-regulated in Med group; 2249 genes were up-regulated, and 2043 genes were down-regulated in High group (Figure 3A–C). The above results suggest that the number of DEGs showed a dose-dependent relationship with the concentration of Hg. As shown in Figure 3D, the Venn diagram depicts overlapping and non-overlapping numbers of DEGs among three comparisons. The Venn diagram demonstrates that 641 genes were all significantly changed in the three exposure groups (Low, Med, and High), indicating that these genes might play important role in Hg exposure. The expressions of the 641 genes were significantly changed under their exposure to Hg in different concentrations (Figure 3E).

### 3.7. GO and KEGG Analyses of DEGs

GO analysis (Table S7) showed that the hormone metabolic process, anion transport, response to drug, drug transmembrane transport, and drug transport were markedly enriched (corrected *p* < 0.05) after exposure to low concentrations of Hg. Moreover, organic acid metabolic processes, carboxylic acid metabolic processes, and small molecule metabolic processes were significantly enriched after exposure to Med concentrations of Hg. Long-chain fatty acid metabolic processes, small molecule metabolic processes, and fatty acid metabolic processes were significantly enriched after exposure to high concentrations of Hg.

**Figure 4:**
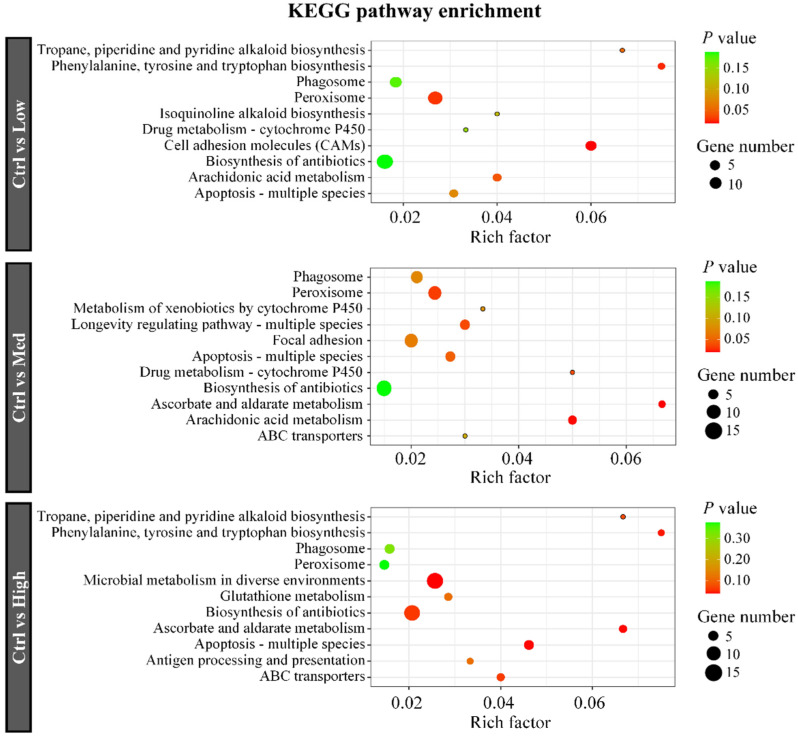
KEGG enrichments of the DEGs after treatment with different concentrations of Hg. The horizontal axis denotes the rich factor while the vertical axis denotes pathways. Color shades represent different *p*-values while dot sizes denote the number of DEGs. A larger dot indicates more DEGs. Ctrl: 0 μg/L Hg^2+^; Low: 8.75 μg/L Hg^2+^; Med: 21.875 μg/L Hg^2+^; High: 43.75 μg/L Hg^2+^.

Analysis of KEGG was conducted to confirm significantly enriched pathways in crayfish after Hg treatment. Each of the ten enriched KEGG pathways in the three comparisons is presented in Figure 4. KEGG enrichment analysis showed the DEGs were enriched in phagosome, peroxisome, apoptosis-multiple species, and biosynthesis of antibiotics signaling pathways in comparisons (Ctrl vs. Low, Ctrl vs. Med, and Ctrl vs. High). Drug metabolism—cytochrome P450, and arachidonic acid metabolism were significantly enriched in comparisons (Ctrl vs. Low, Ctrl vs. Med). Ascorbate and aldarate metabolism and ABC transporters were significantly enriched in comparisons (Ctrl vs. Med, Ctrl vs. High).

**Figure 5:**
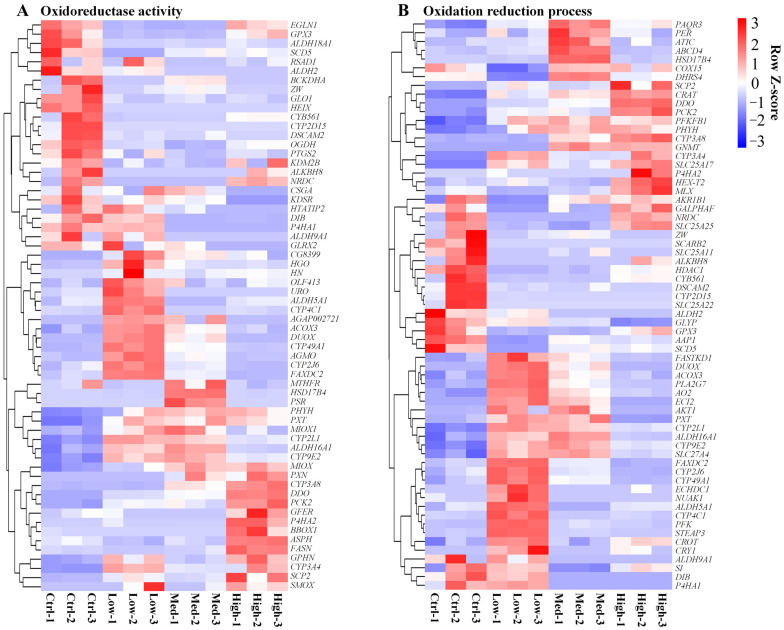
The effects of treatment with different concentrations of Hg on redox metabolism-related gene expression. Terms linked to redox metabolism, such as oxidoreductase activity (**A**) and oxidation-reduction process (**B**). Each gene was assessed based on its average FPKM value. The genes with higher expression levels are colored red and those with lower expression levels are colored blue. Ctrl: 0 μg/L Hg^2+^; Low: 8.75 μg/L Hg^2+^; Med: 21.875 μg/L Hg^2+^; High: 43.75 μg/L Hg^2+^.

**Figure 6:**
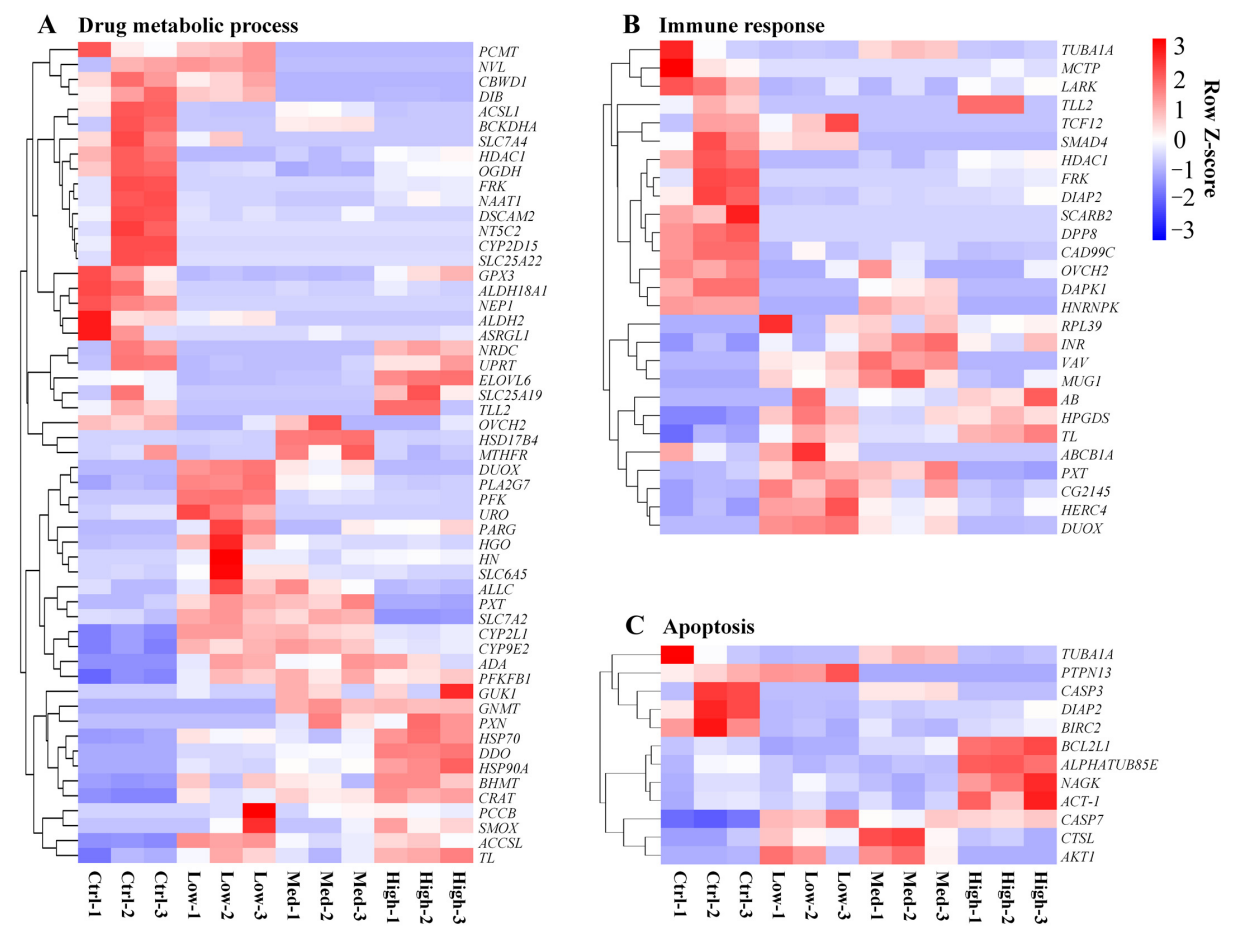
The effects of treatment with different concentrations of Hg on drug metabolism, immune response, and apoptosis-related gene expression. The heat map illustrates variations in drug metabolism (**A**), immune response (**B**), and apoptosis-related gene expression (**C**). Each gene was assessed based on its average FPKM value. The genes with higher expression levels are colored red and those with lower expression levels are colored blue. Ctrl: 0 μg/L Hg^2+^; Low: 8.75 μg/L Hg^2+^; Med: 21.875 μg/L Hg^2+^; High: 43.75 μg/L Hg^2+^.

In particular, the DEGs linked to redox metabolism (oxidoreductase activity and oxidation-reduction process) (Figure 5A,B), ion transport (calcium ion transport, sodium ion transport, potassium ion transport, phosphate ion transport, iron ion transport, zinc ion transport, copper ion transport, and magnesium ion transport) (Figure S5A–I), drug metabolic process (Figure 6A), immune response (Figure 6B), as well as apoptosis (Figure 6C), were analyzed.

### 3.8. qPCR Analysis for Verification of Transcriptome Data

**Figure 7:**
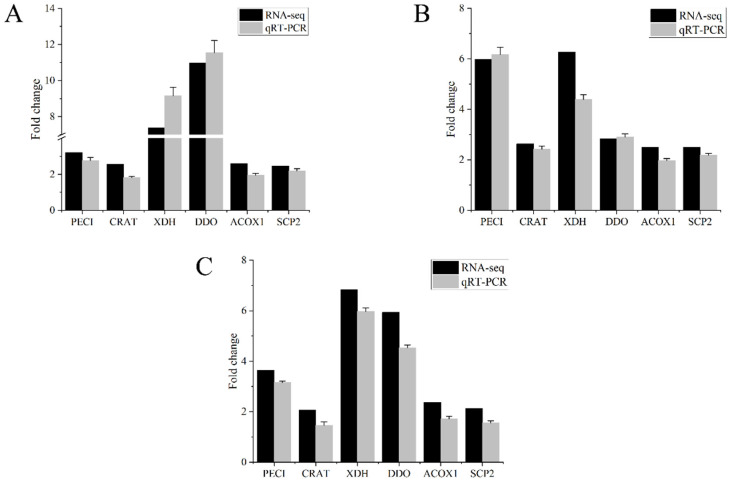
Validation of RNA-seq gene expressions in *P. clarkii* hepatopancreas by qRT-PCR: (**A**) Low concentration of Hg (8.75 μg/L Hg^2+^). (**B**) Med concentration of Hg (21.875 μg/L Hg^2+^). (**C**) High concentration of Hg (43.75 μg/L Hg^2+^).

A total of 641 DEGs were shared under exposure to Hg in different concentrations (Figure 3D). In order to validate the RAN-seq results, six of these shared DEGs, along with being enriched into the peroxisome pathway (Figure S6), including PECI, CRAT, XDH, DDO, ACOX1, and SCP2, were selected for qPCR analyses. As shown in Figure 7, even though there were some variations in fold changes by different computing as well as measuring methods, expression profiles of the chosen DEGs analyzed by qPCR were consistent with the transcriptome data, indicating that the transcriptome data were reliable and accurate. 

### 3.9. Sequencing

After screening for quality, 1,008,924 valid reads were obtained from all crayfish intestinal samples. These valid sequences ranged from 65,704 to 94,362 (Table S8). As displayed in Figure S7A, there was a fast ascension of the species accumulation curve, which reached saturation as the sample number increased, implying that there were enough samples in this assay to fully reflect microbial richness. Moreover, rarefaction curves (Figure S7B) revealed high sampling coverage (>99%) in every sample with sufficient sequencing depth.

### 3.10. Alpha-Diversity, Beta-Diversity, and OTU Distribution

**Figure 8:**
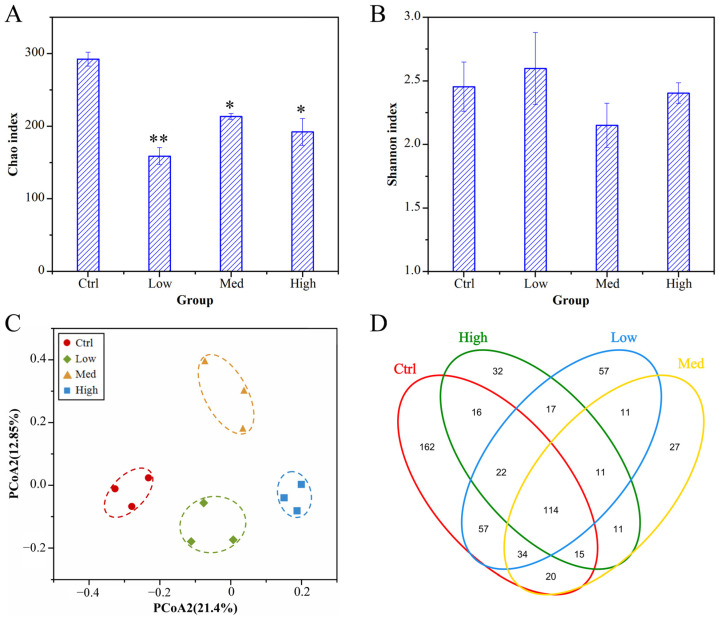
Alpha- and Beta-diversities of intestinal microbiome of *P. clarkii* in the Hg-treated groups: (**A**) Chao indices of bacterial community. (**B**) Shannon indices of bacterial community. (**C**) PCoA of the microbiome. (**D**) Venn diagram showing the number of shared and unshared OTUs among the different Hg-treated groups. * *p* ≤ 0.05, ** *p* ≤ 0.01. Data are shown as mean ± SD. Ctrl: 0 μg/L Hg^2+^; Low: 8.75 μg/L Hg^2+^; Med: 21.875 μg/L Hg^2+^; High: 43.75 μg/L Hg^2+^.

Alpha-diversity indices for the intestinal microbiome in the Ctrl and Hg-treated crayfish are shown in Figure 8A,B and Table S8. The Chao index of *P. clarkii* was decreased significantly in the Hg treatment groups, relative to Ctrl group. However, there was no significant difference in the Shannon index of Hg-treated groups compared with Ctrl group. Based on unweighted UniFrac distance PCoA analyses (Figure 8C), samples from Ctrl and three Hg-treated were divided into four groups. It was found that Hg exerted potential effects on the intestinal microbiota structures of crayfish.

The Venn diagram in Figure 8D shows that 114 OTUs were common in all four groups, suggesting that the OTUs could be insensitive to Hg. In addition, 162 OTUs were limited to the control group, implying that they were sensitive to Hg. In comparison, 57, 27, and 32 OTUs were, respectively, exclusive in Low, Med, and High groups. Thus, we hypothesized that Hg altered the gut microbiome of crayfish.

### 3.11. Intestinal Microbiome Composition

**Figure 9:**
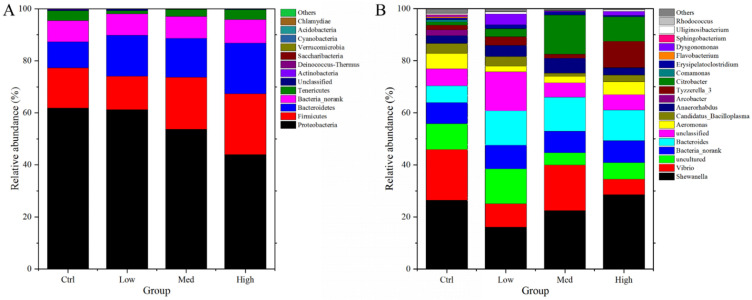
The influences of Hg exposure on intestinal microbiome composition at phyla and genera level: (**A**) Relative abundances of the dominant bacteria at phyla level among the four Hg exposure groups. (**B**) Relative abundances of the dominant bacteria at genera level among the four Hg exposure groups. Ctrl: 0 μg/L Hg^2+^; Low: 8.75 μg/L Hg^2+^; Med: 21.875 μg/L Hg^2+^; High: 43.75 μg/L Hg^2+^.

Figure 9A showed that the most abundant bacterial phyla were *Proteobacteria* (43.97–61.83%), *Firmicutes* (12.90–23.40%), *Bacteroidetes* (10.05–19.43%), and *Tenericutes* (1.23–3.84%) in the intestine of *P. clarkii*. At the genus level (Figure 9B), the top four genera were *Shewanella* (16.08–28.60%), *Vibrio* (5.98–19.45%), *Bacteroides* (6.45–13.28%), and *Aeromonas* (2.50–6.00%). Relative to Ctrl group, the high concentration of Hg markedly decreased *Proteobacteria* abundance, whereas it induced a significant increase in *Firmicutes* (Table S9). The relative abundance of the genera exhibited marked variations in at least one Hg-treated group (Table S10). *Arcobacter* showed a significant decrease in abundance, while *Bacteroides* and *Dysgonomonas* showed a significant increase in abundance. Notably, these changes were primarily significant in the High concentration treatment group.

## 4. Discussion

Hg has previously been evaluated for its toxic effects on crustaceans, and most of these studies were focused on the median lethal concentration (LC50). In these articles, the effect of temperature on the LC50 of Hg, the effect of Hg exposure on ovarian maturation, and a health risk assessment on humans of Hg accumulation in *P. clarkii* were researched [56,72,73]; however, Hg toxicity to crayfish has not been fully established. Therefore, we evaluated histological variations, the hepatopancreatic transcriptome, and intestinal microbiota to establish how *P. clarkii* responds to Hg at biochemical, physiological, tissue, molecular, as well as gut flora levels.

### 4.1. Influence of Hg on Biochemical and Physiological Variations in P. clarkii

In this research, we examined the concentration-dependent uptake of Hg into tissues (gill, antennal gland, hepatopancreas, and abdominal muscle) of *P. clarkii* after aqueous exposure. As a primary organ of detoxification, the hepatopancreas is a major reservoir of xenobiotics and is vulnerable to metal accumulation [74,75]. In our study, with increasing Hg concentrations, the accumulation level of Hg in hepatopancreas significantly increased (*p* < 0.01) after 96 h of exposure. These results are consistent with the above literature that the hepatopancreas of *P. clarkii* could accumulate Hg effectively.

Apoptosis and the death of cells are linked to excessive ROS [76]. MDA is considered a biomarker of oxidative damage and the main mechanism by which ROS induces tissue injury [77]. In this research, increasing Hg concentrations significantly increased ROS and MDA levels, suggesting that Hg exposure can induce oxidative stress and result in membrane polyunsaturated fatty acid peroxidation. Antioxidants play a significant role in regulating ROS levels in cells and preventing oxidative damage to the organism [78]. Biological systems establish a first-line defense against ROS by controlling SOD and CAT activity [77]. CAT is an antioxidant that can protect cells and organs from H_2_O_2_-induced damage [79]. In the present study, a decrease in SOD and CAT activity observed is consistent with ROS being generated in response to Hg. The results are in accordance with the literature that exposure to Hg reduced the SOD and CAT activity in *Scylla serrata* [80]. It has been reported that the superoxide anion radical inhibits CAT activity, which may also explain the lesser activity of CAT [81]. As a tripeptide, GSH stimulates strong antioxidant activity by reversing ROS-induced damage [82]. In our study, as a result of Hg exposure for 96 h, GSH levels in hepatopancreas decreased significantly in a concentration-dependent manner, indicating the rapid and sustained responses of GSH in Hg detoxification. Lower concentrations of GSH are consistent with the greater utilization of GSH in reducing H_2_O_2_ to H_2_O. As a detoxifying enzyme, GST suppresses ROS production [83]. Hg exposure results in higher GST activity, which indicates detoxification mechanisms are activated. There has been evidence that Hg causes oxidative stress in several species [80,84]; as a result, it could be hypothesized that GST’s greater activity in crayfish exposed to Hg is an adaptation to oxidative stress. A dose-dependent increase in ROS levels was observed after MeHg exposure in *Brachionus koreanus* and *Paracyclopina nana* [85]. The GSH levels in *Brachionus koreanus* were also significantly decreased after exposure to 500 and 1000 ng/L MeHg [86]. The above results of MeHg toxicity on ROS and GSH levers coincide with the influence of inorganic Hg^2+^ on *Procambarus clarkii* in our study. 

### 4.2. Influence of Hg on Histological Variations in P. clarkii

In this study, Hg toxicity was assessed by examining the histological alternations of tissues. Hg was associated with various injuries to the hepatopancreas, including epithelium vacuolization and lumen dilatation. Other studies showed that Hg caused severe alterations in the hepatopancreas of prawn *Macrobrachium malcolmsonii* [87] and tropical *Macrobrachium rosenbergii* [88]. In the intestine, after treatment with med and high concentrations of Hg, there was apparent damage to epithelial cells. There were irregular-shaped, abnormal palisade-arranged epithelial cells, implying that Hg damaged the intestinal microvilli of *P. clarkii*. Intestinal histological damage or alterations were also reported in common carp after exposure to Hg [89]. Thus, intestinal abnormalities imply that intestinal structures of *P. clarkii* were damaged by Hg, which might, in turn, lead to variations in the diversity, richness, and composition of the intestinal microbiome.

### 4.3. Influence of Hg on the Hepatopancreatic Transcriptome of P. clarkii

Using hepatopancreatic transcriptome analysis, the influences of Hg on redox metabolism, ion transport, drug metabolism, immune response, as well as apoptosis-associated genes and signaling pathways in *P. clarkii* were revealed. The following are the details of the classified discussion.

#### 4.3.1. Influence of Hg on Redox Metabolism in *P. clarkii*

Redox homeostasis is essential to sustain metabolism and growth [90]. As a result of redox metabolism, excess levels of ROS are removed and cellular redox balance is reestablished [91,92]. The related genes involved in the redox metabolism of *P. clarkii* under exposure to Hg are still little known. In this study, a large number of genes related to oxidoreductase activity (Figure 5A) and the oxidation-reduction process (Figure 5B) were identified to be involved in the molecular regulatory network of *P. clarkii* under the exposure of Hg.

During the peroxisomal beta-oxidation of fatty acids, acyl-CoA oxidase 3 (ACOX3) produces hydrogen peroxide (H_2_O_2_) [93]. Oxidative stress can be induced by H_2_O_2_, a highly reactive oxidant [94]. Spermine oxidase (SMOX) can produce reactive oxygen species (ROS) while degrading polyamines [95], further inducing oxidative stress. During xenobiotic chemical metabolism, cytochrome P450 (CYP) produces oxygen free radicals, which can lead to oxidative stress. In the early stages of exposure to chemicals, P450 is normally expressed [96]. In this study, ACOX3, SMOX, CYP2J6, CYP3A4, CYP3A8, CYP4C1, CYP9E2, and CYP49A1 were found to be significantly up-regulated under single or multiple concentrations of Hg exposure, which indicate that Hg exposure increases the production of ROS and H_2_O_2_. These results provide evidence supporting our findings that Hg exposure significantly increases the production of ROS, further causing oxidative stress in *P. clarkii*.

Molecular antioxidants can prevent oxidative damage to target molecules [97]. As part of the major oxidative pathway that involves alcohol metabolism, aldehyde dehydrogenase (ALDH) plays a significant role [98]. As a result of the activation of ALDH isozymes, reactive aldehydes within cells can be detoxified and oxidative insults from ROS could be prevented [99]. Through the reduction in peroxides, glutathione peroxidase 3 (GPX3) protects cells from oxidative damage [100]. Knockdown of glyoxalase 1 (GLO1) in nondiabetic mice can induce oxidative stress [101]. Additionally, the mammalian GLO1 gene contains an antioxidant-responsive element, which helps GLO1 participate in the major cytoprotective antioxidant system [102]. Through the HIF-1 pathway, Prolyl 4-hydroxylase subunit alpha 1 (P4HA1) can inhibit oxidative phosphorylation and ROS generation [103]. In our study, these antioxidant-related genes include ALDH2, ALDH9A1, ALDH18A1, GPX3, GLO1, as well as P4HA1, which were found to be significantly down-regulated under single or multiple concentrations of Hg exposure. These results indicate that Hg exposure decreases the antioxidant production related to these genes, further perturbing the redox equilibrium of *P. clarkii*.

#### 4.3.2. Influence of Hg on Ion Transport in *P. clarkii*

A number of crucial physiological parameters are controlled by ion transport, including ion balance and membrane potential, which are prerequisites for regulating the vital functions in life processes [104,105]. In this study, abundant genes related to ion transport (Figure S5) were identified to be involved in the molecular regulatory network of *P. clarkii* under the exposure of Hg.

Aspartyl/asparaginyl beta-hydroxylase (ASPH) regulates calcium (Ca) cycling in cardiomyocytes, knockout ASPH in mice exhibits impaired fertility, morphological defects, and abnormal heart function [106,107]. Calcium uptake protein 3 (MICU3) can increase mitochondrial Ca^2+^ uptake dramatically. Multiple C2 and transmembrane domain-containing protein (MCTP) stabilize baseline calcium release by acting downstream of calcium influx [108]. In our study, ASPH was significantly changed, MICU3 was significantly upregulated in Med and High groups, and MCTP was downregulated in all Hg-treated groups; these above results indicated that Hg exposure showed a stronger effect on the calcium cycling, mitochondrial Ca^2+^ uptake, and calcium influx.

In the liver and intestine, solute carrier family 10 member 2 (SLC10A2) encodes a sodium-dependent bile acid transporter [109]. As a result of pyrexia (PYX) activation, potassium will be effluxed from the cell [110]. The STEAP3 metalloreductase (STEAP3) converts copper and iron from trivalent to divalent cationic forms and maintains homeostasis [111]. Tumor Suppressor Candidate 3 (TUSC3) plays a role in embryonic development, protein glycosylation, and cellular magnesium uptake [112]. Zinc transporter 1 is encoded by the soluble carrier family 39 member 1 gene (SLC39A1). Studies have shown that SLC39A1 is overexpressed in prostate cancer, causing depletion of zinc in the glands [113,114]. Zinc transporter ZnT2 is also known as SLC30A2 (Solute carrier family 30 member 2) and is crucial for transporting zinc into the mammary epithelium [115]. The over-expression of SLC30A2 results in Zn vesicularization, reduced proliferation, and enhanced apoptosis [116]. In this study, these prominent changes in genes, including SLC10A2, PYX, STEAP3, TUSC3, SLC39A1, and SLC30A2, indicate that Hg exposure has a negative influence on the ion transport of sodium, potassium, iron, copper, magnesium, as well as zinc, and may well disrupt the ion homeostasis of *P. clarkii*. In addition, the significant up-regulation of SLC30A2 with rising Hg concentrations suggest that Hg exposure may cause apoptosis via Zn accumulation in hepatopancreas cell.

#### 4.3.3. Influence of Hg on Drug Metabolism in *P. clarkii*

In response to Hg toxicity, a large number of drug metabolism-related DEGs were identified (Figure 6A). The heat shock proteins (HSPs), also known as “stress proteins”, play an important role in protecting the body from environmental and cellular stress [117,118]. Moreover, HSPs can protect against oxidative stress [119]. In the current study, the up-regulation of HSP70 and HSP90A may protect hepatopancreas cells against the oxidative stress and protein damage induced by Hg exposure. In addition, CYPs are involved in exogenous substance detoxification and are a potential biomarker for evaluating pollutants in aquatic environments [120]. The influence of Hg on CYPs is less studied in crustaceans. In mouse, Hg was shown to up-regulate Cyp1a1, Cyp2b9, Cyp2b10, Cyp2b19, Cyp4a10, Cyp4a12, and Cyp4a14 genes [121]. We found up-regulations of CYP2L1 and CYP9E2 in *P. clarkii* after exposure to Hg, which suggests that they play an important role in the detoxification of Hg.

#### 4.3.4. Influence of Hg on Immune Response in *P. clarkii*

In crustaceans, the hepatopancreas is a critical immune organ responsible for regulating metabolic processes and immune responses [122]. In our study, several genes related to immune response (Figure 6B) were identified as belonging to the hepatopancreas in *P. clarkii* under Hg exposure. Loss or deficiency of SMAD family member 4 (SMAD4) in T cells often causes immune diseases [123]. By binding to the NF-kB co-repressor, histone deacetylase 1 (HDAC1) has a wide range of effects on the immune system in mammals [124]. DUOX (dual oxidase) suppression promotes effective immune responses, leading to lower infection loads [125]. After Hg exposure, the SMAD4 and HDAC1 were down-regulated and DUOX was up-regulated in our study; these results suggest that Hg exposure could weaken the immune response and ability, as well as induce immune diseases.

#### 4.3.5. Influence of Hg on Apoptosis in *P. clarkii*

Apoptosis is important in homeostasis maintenance and in aquatic organisms; it is initiated by several environmental stressors [126]. Hg was shown to initiate apoptosis in various cell types, such as grass carp cell ZC7901 and marine teleost fish SAF-1 cell lines [127,128]. In our study, expressions of various apoptosis-associated genes, such as cathepsin L (CTSL), baculoviral IAP repeat containing 2 (BIRC2), and caspase 7 (CASP7) were markedly altered in the *P. clarkii* hepatopancreas after Hg exposure. As a cysteine protease, CTSL is highly involved in various apoptosis-associated pathways [129]. Elevated CTSL induces apoptosis by the release of cytochrome c from the mitochondria and the activation of mitochondrial apoptosis [130,131]. BIRC2 belongs to the inhibitor of the apoptosis family of antiapoptotic proteins. Apoptosis inhibitors suppress apoptosis by downregulating procaspase activation, as well as by directly suppressing activated caspases [132]. CASP7 has been shown to be an important executioner protein of apoptosis [133]. In our study, the expression of CTSL and CASP7 was overall increased and the expression of BIRC2 was decreased under Hg exposure. These results indicate that apoptosis occurred in *P. clarkii* hepatopancreas after acute exposure to Hg. Furthermore, the obvious tissue damage in the hepatopancreas caused by Hg exposure may be mediated by the activation of apoptosis.

Integrating these above findings, we speculate a potential mechanism of how Hg exposure damages crustaceans’ tissues. Our results lead to the following deduction, exposure to inorganic Hg^2+^ induces apoptosis via oxidative stress and the dysregulation of ion homeostasis, further causing tissue damage in crustaceans. On the one hand, exposure of *P. clarkii* to Hg^2+^ significantly increases ROS and MDA levels and inhibits the activity of SOD and CAT, further inducing oxidative stress and apoptosis. On the other hand, exposure to Hg^2+^ downregulates MCTP expression and enhances Ca^2+^ influx, upregulating SLC30A2 and causing Zn vesicularization, further triggering apoptosis. However, this deduction needs to be confirmed by further study.

### 4.4. Effect of Hg on Intestinal Microbiota in P. clarkii

The intestine is a complex micro-ecosystem, in which inhabits a huge and varied microbial community consisting of archaea, viruses, bacteria, and various unknown eukaryotes. Intestinal histopathology, morphology, and microbiota balance are important indicators of intestinal health [134]. In our study, Hg exposure damaged the intestine resulting in microvilli vacuolization and extended lamina proprias, which might lead to variations in the diversity, richness, and composition of the intestinal microbiota. 

The physiological status of the host, especially the immune system, is closely related to the intestinal microbiota [135,136]. In this research, Hg exposure dysregulated the intestinal microbiota in *P. clarkii*. The decreased ACE and Chao indices in Hg-treated groups showed that intestinal microbiota exposed to Hg had a significantly low abundance (*p* < 0.05). Based on PCoA plots, samples were allocated into four parts, implying that Hg dysregulated the intestinal microbiome.

In this study, *Firmicutes*, *Proteobacteria*, and *Bacteroidetes* were established to be the dominant phyla in the intestines of *P. clarkii*. These findings are in tandem with those found in other aquatic crustaceans [137,138,139]. Thus, we concluded that these three phyla are predominant in the gut of aquatic crustaceans. *Proteobacteria* plays various roles in several biogeochemical processes, such as carbon, sulfur, and nitrogen cycling [140]. A high abundance of *Firmicutes* enhances fatty acid absorption [141]. *Bacteroidetes* are involved in the cycling of protein-rich substances as well as complex carbon [142]. In this study, *Proteobacteria*, *Firmicutes*, as well as *Bacteroidetes* accounted for over 86.80% of the total phyla in the gut of *P. clarkii*, implying that they are of significance in digestion as well as absorption. Hence, the considerable decrease in *Proteobacteria* and increase in *Firmicutes* suggest that carbon, sulfur, and nitrogen cycling were weakened, while fatty acid absorption was promoted in the gut of *P. clarkii* after exposure to high concentrations of mercury. 

*Bacteroides* secretes various capsular polysaccharides to change its surface antigenicity in the human colon [143]. It can regulate the milieu via interactions with host immune systems to control the proliferation of other bacteria [144]. *Bacteroides* has an abundance of enzymes involved in carbohydrate transport as well as protein metabolism. Moreover, it has vitamins, glycans, and co-factor enzymes, which are important in digestion [145]. Thus, a high intestinal abundance of this genus implied an adaptation of *P. clarkii* to Hg. *Dysgonomonas macrotermitis* is the only *Dysgonomonas* species in invertebrates, and its major role in hind-intestines of *Macrotermes barneyi* is to decompose lignocellulose and provide nutrition to its host [146]. The high *Dysgonomonas* abundance indicates that Hg exposure enhances the digestion of plant raw materials by *P. clarkii*. Meanwhile, there were also some pathogenic bacteria, such as *Arcobacter* in the gut of Hg-exposed *P. clarkii*. Some *Arcobacter* species induce infections in animals and humans, and they are also considered zoonotic and enteropathogenic [147]. Taken together, Hg exposure damages the intestine tissue, reduces the abundance of intestinal microbiota, and dysregulates the intestinal microbiota composition, which suggests that Hg exposure can further affect the overall health of *P. clarkii*.

## 5. Conclusions

In this study, Hg accumulated in the hepatopancreas in a dose-dependent manner. Oxidative stress and antioxidant parameters, including ROS, MDA, SOD, CAT, GSH, and GST, showed significant variations after being challenged with Hg in the hepatopancreas. Hence, these parameters might serve as effective, rapid, and sensitive biomonitoring parameters of mercury. Additionally, Hg exposure caused tissue damage to the hepatopancreas and intestine, and the degree of tissue damage was shown to be dose-dependent. Using transcriptomes analysis, we identified a lot of redox metabolism, ion transport, drug metabolism, immune response, as well as apoptosis-associated DEGs under Hg exposure. Based on these genetic alterations, we clarified the potential mechanism involved in the enhanced production of ROS in Hg exposure and speculated that Hg exposure may perturb the redox equilibrium, disrupt the ion homeostasis, weaken immune response and ability, and cause apoptosis. Meanwhile, Hg exposure decreased microbiome richness, and markedly altered the microbial structure in the intestines of crayfish at the phylum as well as the genus level. As a result of this study, we gained valuable insight into the toxic mechanisms of heavy metals in crayfish. Moreover, the insights gained from this study will be useful for future research and the assessment of potential antioxidant and immunomodulatory effects in polluted environments and crustacean farming.

## Data Availability

Data are contained within the article and Supplementary Materials.

## Supplementary Materials

The following supporting information can be downloaded at: https://www.mdpi.com/article/10.3390/antiox11101944/s1, Figure S1: Quantified results of the hepatopancreas tubule lumen dilatation (A) and intestine microvilli vacuolization (B) proportion in the Ctrl, Low, Med, and High groups (mean ± SD, n = 9). Figure S2: Length distribution of *P. clarkii* hepatopancreas unigenes. Figure S3: Classification of assembled unigenes. GO function classification (A–C), KOG classification histogram presentation (D), and KEGG classification (E) of assembled unigenes. Figure S4: Principal Component Analysis (PCA) of the gene expression of samples. Figure S5: The heat map illustrates variations in ion transport-related gene expression. Figure S6: Peroxisome pathway of *P. clarkii* induced by high concentration of Hg. Figure S7: (A) Species accumulation curves of *P. clarkii* intestinal microbial samples. (B) Rarefaction curves of all the samples based on Illumina MiSeq sequencing. Table S1: Primers used in the quantitative PCR analysis. Table S2: Measured and nominal Hg concentrations in water during the exposure experiment. Table S3: Bioaccumulation of Hg in tissues during exposure to different concentrations of Hg. Table S4: Effect of different concentrations of Hg on oxidative stress and antioxidant parameters in hepatopancreas of *P. clarkii*. Table S5: Statistics of hepatopancreas transcriptome sequencing. Table S6: Summary of the annotations. Table S7: GO enrichment analysis of DEGs. Table S8: Richness and diversity indices of bacterial communities for all intestinal samples. Table S9: The microbial composition (mean ± SE) of *P. clarkii* after exposure to different concentrations of Hg at the phylum level. Table S10: The microbial composition (mean ± SE) of *P. clarkii* after exposure to different concentrations of Hg at the genus level.
