# Supplementary material for: Mercury Induced Tissue Damage, Redox Metabolism, Ion Transport, Apoptosis, and Intestinal Microbiota Change in Red Swamp Crayfish (Procambarus clarkii): Application of Multi-Omics Analysis in Risk Assessment of Hg
Source: Antioxidants (Basel). 2022 Sep 29;11(10):1944. doi: 10.3390/antiox11101944 (PMC9598479; doi:10.3390/antiox11101944)
Supplement: Supplementary file 1 [file antioxidants-11-01944-s001.zip › Figure S1.pdf]

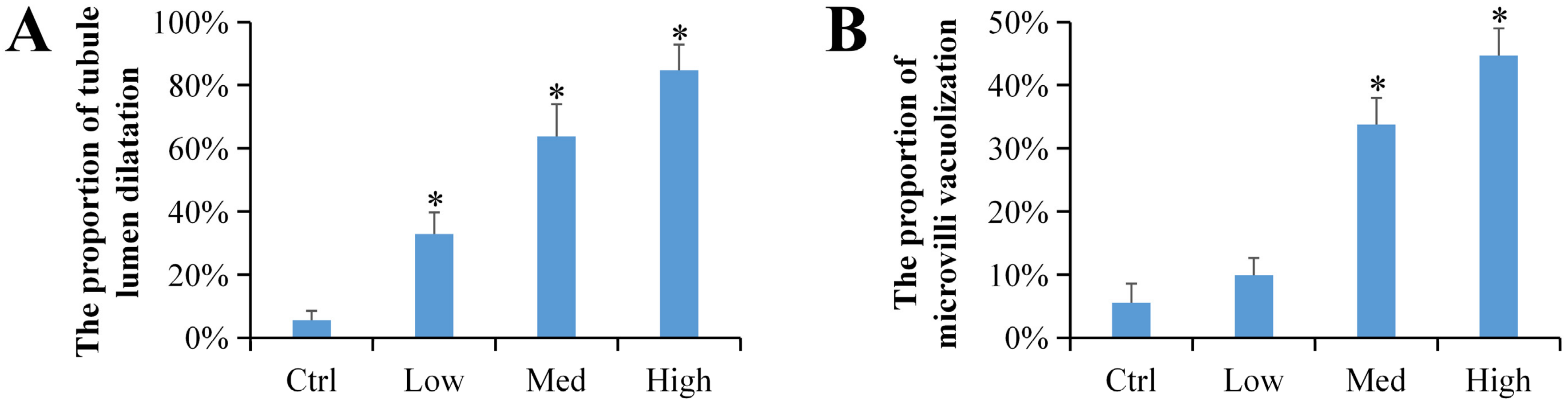

Figure S1. Quantified results of the hepatopancreas tubule lumen dilatation (A) and intestine microvilli vacuolization (B) proportion in the Ctrl, Low, Med, and High groups (mean ± SD, n = 9). *, $P < 0.05$.

.
