# Supplementary material for: Mercury Induced Tissue Damage, Redox Metabolism, Ion Transport, Apoptosis, and Intestinal Microbiota Change in Red Swamp Crayfish (Procambarus clarkii): Application of Multi-Omics Analysis in Risk Assessment of Hg
Source: Antioxidants (Basel). 2022 Sep 29;11(10):1944. doi: 10.3390/antiox11101944 (PMC9598479; doi:10.3390/antiox11101944)
Supplement: Supplementary file 1 [file antioxidants-11-01944-s001.zip › Figure S3.pdf]

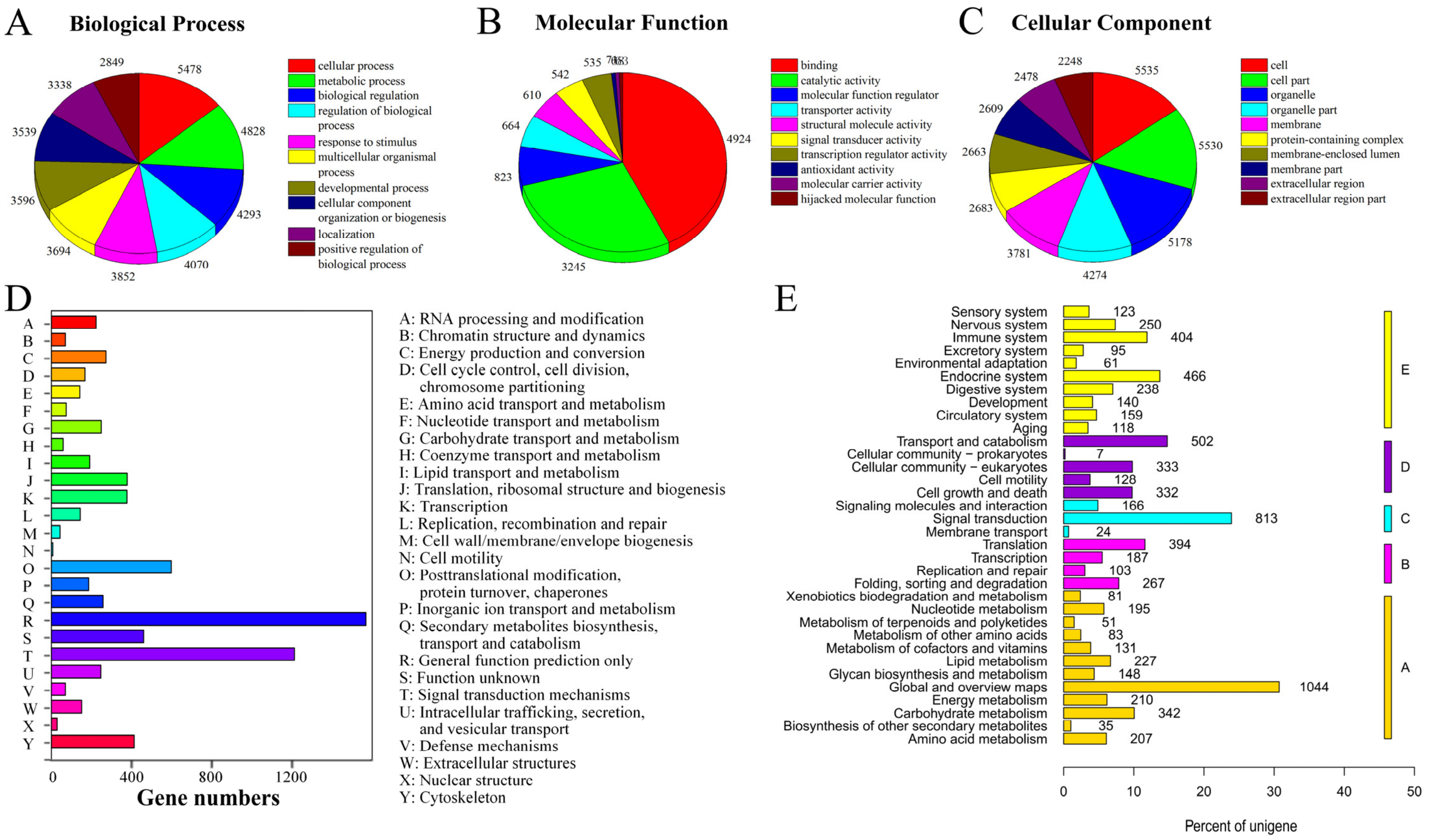

Figure S3. Classification of assembled unigenes. The GO function classification results were classified into three categories: Biological Process (A), Molecular Function (B), and Cellular Component (C). KOG classification histogram presentation (D) and KEGG (E) classification of assembled unigenes.
