# Supplementary material for: Mercury Induced Tissue Damage, Redox Metabolism, Ion Transport, Apoptosis, and Intestinal Microbiota Change in Red Swamp Crayfish (Procambarus clarkii): Application of Multi-Omics Analysis in Risk Assessment of Hg
Source: Antioxidants (Basel). 2022 Sep 29;11(10):1944. doi: 10.3390/antiox11101944 (PMC9598479; doi:10.3390/antiox11101944)
Supplement: Supplementary file 1 [file antioxidants-11-01944-s001.zip › Figure S5.pdf]

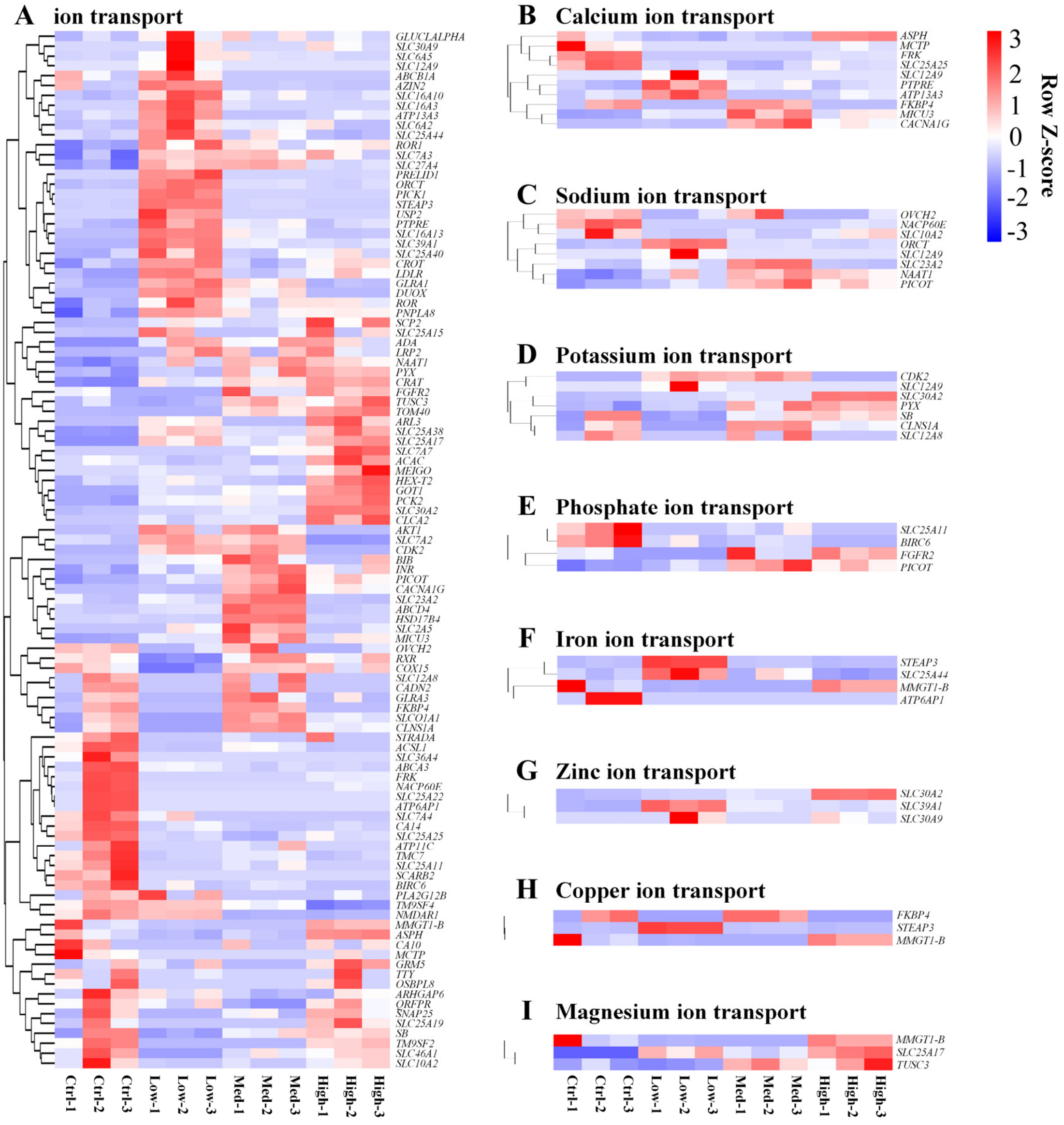

Figure S5. The heat map illustrates variations in ion transport-related gene expression. Terms linked to ion transport, like ion transport (A), calcium ion transport (B), sodium ion transport (C), potassium ion transport (D), phosphate ion transport (E), iron ion transport (F), zinc ion transport (G), copper ion transport (H), and magnesium ion transport (I). Each gene was assessed based on its average FPKM value. The genes with higher expression levels are colored red and those with lower expression levels are colored blue.
