# Supplementary material for: Mercury Induced Tissue Damage, Redox Metabolism, Ion Transport, Apoptosis, and Intestinal Microbiota Change in Red Swamp Crayfish (Procambarus clarkii): Application of Multi-Omics Analysis in Risk Assessment of Hg
Source: Antioxidants (Basel). 2022 Sep 29;11(10):1944. doi: 10.3390/antiox11101944 (PMC9598479; doi:10.3390/antiox11101944)
Supplement: Supplementary file 1 [file antioxidants-11-01944-s001.zip › Table S1.pdf]

Table S1 Primers used in the quantitative PCR analysis.

Gene abbreviation	Sequence ID	Forward primers (5’-3’)	Reverse primers (5’-3’)	Amplicon size (bp)	Efficiency (%)
PECI	TRINITY_DN752_c0_g1	AAGCCGCAGAAGATCCCAACAC	ACAAACCGCTCCAGCAGAACTC	154	94.3
CRAT	TRINITY_DN908_c1_g2	CCATTCGCTCCTGTTCGGTTGA	GCCAGTTTCAGCCCAAGAAGGT	178	102.6
XDH	TRINITY_DN3837_c2_g1	GCGGGTGTTTACAGTGCTTTGC	CAGTGGCGACTCCATCGTTACG	165	104.7
DDO	TRINITY_DN9185_c2_g2	TGCGAGTGCTGGCGTCAAAC	TGTGGTGGTGAAGGCGGAGTT	140	93.1
ACOX1	TRINITY_DN1613_c0_g4	GTGCTGACCTGCTTCCTGATGC	TGCCAGTGCTTGGATCGTGTTG	163	96.8
SCP2	TRINITY_DN17174_c1_g3	ACCTGTGGTCAGCGTGCCTT	CTGCCCTCCCAAGACAAGATTGC	126	106.7
18S rRNA	Zhang et al. (2019)	CTGTGATGCCCTTAGATGTT	GCGAGGGGTAGAACATCCAA	/	/

Abbreviations: PECI, Peroxisomal 3,2-trans-enoyl-CoA isomerase; CRAT, carnitine O-acetyltransferase; XDH, xanthine dehydrogenase; DDO, D-aspartate oxidase; ACOX1, acyl-CoA oxidase 1, palmitoyl; SCP2, sterol carrier protein 2; 18S rRNA, 18S ribosomal RNA.
