# Supplementary material for: Mercury Induced Tissue Damage, Redox Metabolism, Ion Transport, Apoptosis, and Intestinal Microbiota Change in Red Swamp Crayfish (Procambarus clarkii): Application of Multi-Omics Analysis in Risk Assessment of Hg
Source: Antioxidants (Basel). 2022 Sep 29;11(10):1944. doi: 10.3390/antiox11101944 (PMC9598479; doi:10.3390/antiox11101944)
Supplement: Supplementary file 1 [file antioxidants-11-01944-s001.zip › Table S10.pdf]

Table S10 The microbial composition (mean ± SE) of *P. clarkii* after exposure to different concentrations of Hg at the genus level.

**Genus**	Relative abundance (%)			
	0 μg/L Hg	8.75 μg/L Hg	21.875 μg/L Hg	43.75 μg/L Hg
Bacteroides	6.46 ± 2.45	13.28 ± 4.59	13.03 ± 3.78*	11.79 ± 1.52*
Dysgonomonas	0.42 ± 0.14	4.14 ± 2.17*	0.32 ± 0.13	1.62 ± 0.17*
Arcobacter	2.26 ± 0.45	0.02 ± 0.01*	0.03 ± 0.01*	0.16 ± 0.03*

Note: *$P \leq 0.05$.
