# Supplementary material for: Mercury Induced Tissue Damage, Redox Metabolism, Ion Transport, Apoptosis, and Intestinal Microbiota Change in Red Swamp Crayfish (Procambarus clarkii): Application of Multi-Omics Analysis in Risk Assessment of Hg
Source: Antioxidants (Basel). 2022 Sep 29;11(10):1944. doi: 10.3390/antiox11101944 (PMC9598479; doi:10.3390/antiox11101944)
Supplement: Supplementary file 1 [file antioxidants-11-01944-s001.zip › Table S2.pdf]

Table S2 Measured and nominal Hg concentrations in water during the exposure experiment.

Group	Nominal concentration (μg/L)	Measured Hg concentrations in water (μg/L)	
		0 h	96 h
Control	0	0	0
Low	8.75	9.03 ± 0.12	8.56 ± 0.16
Med	21.875	22.42 ± 0.07	21.39 ± 0.42
High	43.75	45.04 ± 0.31	43.72 ± 0.84

Note: The expressed values were mean ± SD, n = 3.
