# Supplementary material for: Mercury Induced Tissue Damage, Redox Metabolism, Ion Transport, Apoptosis, and Intestinal Microbiota Change in Red Swamp Crayfish (Procambarus clarkii): Application of Multi-Omics Analysis in Risk Assessment of Hg
Source: Antioxidants (Basel). 2022 Sep 29;11(10):1944. doi: 10.3390/antiox11101944 (PMC9598479; doi:10.3390/antiox11101944)
Supplement: Supplementary file 1 [file antioxidants-11-01944-s001.zip › Table S3.pdf]

Table S3 Bioaccumulation of Hg in tissues during exposure to different concentrations of Hg.

Tissues	Hg bioaccumulation in tissues (μg/g dry weight)			
	Control (0 μg/L Hg)	Low (8.75 μg/L Hg)	Med (21.875 μg/L Hg)	High (43.75 μg/L Hg)
Gill	0.18 ± 0.06	27.69 ± 8.54**	42.51 ± 13.13**	61.27 ± 23.73**
Antennal gland	0.03 ± 0.01	8.78 ± 5.12*	19.76 ± 7.77**	34.73 ± 12.43**
Hepatopancreas	0.08 ± 0.03	7.74 ± 3.13**	12.67 ± 4.76**	18.63 ± 6.15**
Abdominal muscle	0.04 ± 0.01	0.42 ± 0.24*	0.76 ± 0.24**	1.07 ± 0.48**

Note: The expressed values were mean ± SD, n = 3. One asterisk indicates significant differences ($P < 0.05$) and two asterisk indicates highly significant differences ($P < 0.01$) compared to control (0 μg/L Hg) based on different exposure concentrations.
