# Supplementary material for: Mercury Induced Tissue Damage, Redox Metabolism, Ion Transport, Apoptosis, and Intestinal Microbiota Change in Red Swamp Crayfish (Procambarus clarkii): Application of Multi-Omics Analysis in Risk Assessment of Hg
Source: Antioxidants (Basel). 2022 Sep 29;11(10):1944. doi: 10.3390/antiox11101944 (PMC9598479; doi:10.3390/antiox11101944)
Supplement: Supplementary file 1 [file antioxidants-11-01944-s001.zip › Table S4.pdf]

**Table S4 Effect of different concentrations of Hg on oxidative stress and antioxidant parameters in hepatopancreas of *P. clarkii*.**

| Parameter | Test groups | | | |
|---|---|---|---|---|
| | Control (0 μg/L Hg) | Low (8.75 μg/L Hg) | Med (21.875 μg/L Hg) | High (43.75 μg/L Hg) |
| ROS (nmol DCF/mg protein) | 101.64 ± 10.17 | 139.47 ± 12.42* | 143.56 ± 18.73* | 159.78 ± 16.53* |
| MDA (nmol/mg protein) | 2.23 ± 0.15 | 2.76 ± 0.27* | 2.89 ± 0.24* | 3.06 ± 0.31** |
| SOD (U/mg protein) | 112.20 ± 18.63 | 97.68 ± 21.56 | 87.44 ± 16.23 | 76.53 ± 14.65* |
| CAT (U/mg protein) | 38.48 ± 4.27 | 27.57 ± 2.12* | 25.49 ± 3.16* | 21.34 ± 5.66* |
| GSH (μmol/g proterin) | 1870.26 ± 153.89 | 1543.85 ± 179.51* | 1426.42 ± 162.64* | 1367.96 ± 149.54** |
| GST (U/mg protein) | 62.71 ± 4.26 | 71.79 ± 5.34 | 74.81 ± 4.74* | 79.30 ± 6.87* |

**Note:** The expressed values were mean ± SD, n = 3. One asterisk indicates significant differences ($P < 0.05$) and two asterisk indicates highly significant differences ($P < 0.01$) compared to control (0 μg/L Hg) based on different exposure concentrations.
