# Supplementary material for: Mercury Induced Tissue Damage, Redox Metabolism, Ion Transport, Apoptosis, and Intestinal Microbiota Change in Red Swamp Crayfish (Procambarus clarkii): Application of Multi-Omics Analysis in Risk Assessment of Hg
Source: Antioxidants (Basel). 2022 Sep 29;11(10):1944. doi: 10.3390/antiox11101944 (PMC9598479; doi:10.3390/antiox11101944)
Supplement: Supplementary file 1 [file antioxidants-11-01944-s001.zip › Table S5.pdf]

Table S5 Statistics of hepatopancreas transcriptome sequencing.

Sample	Raw Reads	Clean Reads	Clean Bases	Error (%)	Q20(%)	Q30(%)	GC Content(%)
Ctrl_1	50,588,168	50,407,504	7,536,644,750	0.03	97.54	93.72	44.78
Ctrl_2	43,850,794	43,716,022	6,529,828,052	0.03	97.50	93.52	44.49
Ctrl_3	42,795,604	42,694,670	6,380,479,463	0.03	97.45	93.47	44.86
Low_1	56,666,680	56,509,980	8,440,358,084	0.03	97.57	93.82	45.42
Low_2	61,860,116	61,735,916	9,231,380,056	0.03	97.67	93.94	45.60
Low_3	50,141,290	50,010,448	7,475,169,127	0.03	97.55	93.73	45.15
Med_1	49,707,042	49,588,992	7,421,455,320	0.03	97.72	93.99	45.99
Med_2	50,125,034	50,016,118	7,487,179,324	0.03	97.53	93.54	45.88
Med_3	51,623,838	51,531,692	7,722,363,751	0.03	97.48	93.41	45.84
High_1	50,293,278	50,152,158	7,485,105,484	0.03	97.83	94.21	46.54
High_2	50,270,496	50,165,420	7,508,885,766	0.03	97.84	94.24	46.68
High_3	46,462,574	46,325,308	6,935,439,475	0.03	97.65	93.90	46.10
