# Supplementary material for: Mercury Induced Tissue Damage, Redox Metabolism, Ion Transport, Apoptosis, and Intestinal Microbiota Change in Red Swamp Crayfish (Procambarus clarkii): Application of Multi-Omics Analysis in Risk Assessment of Hg
Source: Antioxidants (Basel). 2022 Sep 29;11(10):1944. doi: 10.3390/antiox11101944 (PMC9598479; doi:10.3390/antiox11101944)
Supplement: Supplementary file 1 [file antioxidants-11-01944-s001.zip › Table S6.pdf]

Table S6 Summary of the annotations.

Annotated databases	Number of Unigenes
Annotated in GO	6006
Annotated in KO	5091
Annotated in NR	11564
Annotated in PFAM	5357
Annotated in STRING	7694
Annotated in SWISSPROT	6068
Annotated in KOG	6409
Total Unigenes	192666
