# Supplementary material for: Mercury Induced Tissue Damage, Redox Metabolism, Ion Transport, Apoptosis, and Intestinal Microbiota Change in Red Swamp Crayfish (Procambarus clarkii): Application of Multi-Omics Analysis in Risk Assessment of Hg
Source: Antioxidants (Basel). 2022 Sep 29;11(10):1944. doi: 10.3390/antiox11101944 (PMC9598479; doi:10.3390/antiox11101944)
Supplement: Supplementary file 1 [file antioxidants-11-01944-s001.zip › Table S7.pdf]

Table S7 GO enrichment analysis of DEGs.

GO ID	Description	GO category	Corrected *P*-Value
**Ctrl vs Low**			
GO:0042445	hormone metabolic process	BP	0.0001
GO:0006820	anion transport	BP	0.0003
GO:0009636	response to toxic substance	BP	0.0036
GO:0042493	response to drug	BP	0.0036
GO:0016021	integral component of membrane	CC	0.0059
GO:0015696	ammonium transport	BP	0.0084
GO:0038023	signaling receptor activity	MF	0.0091
GO:0015238	drug transmembrane transporter activity	MF	0.0142
GO:0015893	drug transport	BP	0.0171
GO:0006855	drug transmembrane transport	BP	0.0179
**Ctrl vs Med**			
GO:0006082	organic acid metabolic process	BP	0.0025
GO:0019752	carboxylic acid metabolic process	BP	0.0025
GO:0044281	small molecule metabolic process	BP	0.0025
GO:0043436	oxoacid metabolic process	BP	0.0025
GO:0097305	response to alcohol	BP	0.0051
GO:0030258	lipid modification	BP	0.0166
GO:0015711	organic anion transport	BP	0.0166
GO:0045471	response to ethanol	BP	0.0166
GO:0006820	anion transport	BP	0.0289
GO:0015179	L-amino acid transmembrane transporter activity	MF	0.0404
**Ctrl vs High**			
GO:0001676	long-chain fatty acid metabolic process	BP	0.0031
GO:0044281	small molecule metabolic process	BP	0.0032
GO:0006631	fatty acid metabolic process	BP	0.0034
GO:0032787	monocarboxylic acid metabolic process	BP	0.0034
GO:0019752	carboxylic acid metabolic process	BP	0.0046
GO:0006082	organic acid metabolic process	BP	0.0073
GO:0043436	oxoacid metabolic process	BP	0.0098
GO:0030851	granulocyte differentiation	BP	0.0317
GO:0033993	response to lipid	BP	0.0391
GO:0052689	carboxylic ester hydrolase activity	MF	0.0472

Note: Biological process (BP), cellular component (CC) and molecular function (MF).
