# Supplementary material for: Mercury Induced Tissue Damage, Redox Metabolism, Ion Transport, Apoptosis, and Intestinal Microbiota Change in Red Swamp Crayfish (Procambarus clarkii): Application of Multi-Omics Analysis in Risk Assessment of Hg
Source: Antioxidants (Basel). 2022 Sep 29;11(10):1944. doi: 10.3390/antiox11101944 (PMC9598479; doi:10.3390/antiox11101944)
Supplement: Supplementary file 1 [file antioxidants-11-01944-s001.zip › Table S8.pdf]

Table S8 Richness and diversity indices of bacterial communities for all intestinal samples.

Samples	Seq_num	OTU_num	Shannon_index	ACE_index	Chao1_index	Coverage	Simpson
Ctrl_1	93118	257	2.67	300	282	0.999442	0.1272
Ctrl_2	75970	279	2.2	295	305	0.999566	0.194
Ctrl_3	88870	262	2.49	298	290	0.999415	0.1307
Low_1	80650	151	2.84	190	168	0.999566	0.1047
Low_2	68317	145	2.75	172	166	0.999546	0.0893
Low_3	65704	122	2.2	153	142	0.999498	0.1494
Med_1	94362	168	2.05	224	218	0.999417	0.2771
Med_2	88109	201	2.08	213	208	0.999728	0.3531
Med_3	92287	171	2.32	219	214	0.999491	0.1812
High_1	84183	143	2.51	188	179	0.999501	0.1279
High_2	86972	162	2.39	190	179	0.999621	0.1758
High_3	90382	157	2.31	251	218	0.999447	0.1759
