# Supplementary material for: Mercury Induced Tissue Damage, Redox Metabolism, Ion Transport, Apoptosis, and Intestinal Microbiota Change in Red Swamp Crayfish (Procambarus clarkii): Application of Multi-Omics Analysis in Risk Assessment of Hg
Source: Antioxidants (Basel). 2022 Sep 29;11(10):1944. doi: 10.3390/antiox11101944 (PMC9598479; doi:10.3390/antiox11101944)
Supplement: Supplementary file 1 [file antioxidants-11-01944-s001.zip › Table S9.pdf]

Table S9 The microbial composition (mean ± SE) of *P. clarkii* after exposure to different concentrations of Hg at the phylum level.

**Phylum**	Relative abundance (%)			
	0 μg/L Hg	8.75 μg/L Hg	21.875 μg/L Hg	43.75 μg/L Hg
Proteobacteria	61.83 ± 9.27	61.19 ± 28.04	53.68 ± 15.83	43.97 ± 2.47*
Firmicutes	15.44 ± 2.69	12.90 ± 6.93	20.02 ± 3.42	23.40 ± 0.81*

Note: *$P \leq 0.05$.
