# Supplementary figures and images for: Mercury Induced Tissue Damage, Redox Metabolism, Ion Transport, Apoptosis, and Intestinal Microbiota Change in Red Swamp Crayfish (Procambarus clarkii): Application of Multi-Omics Analysis in Risk Assessment of Hg
Source: Antioxidants (Basel). 2022 Sep 29;11(10):1944. doi: 10.3390/antiox11101944 (PMC9598479; doi:10.3390/antiox11101944)

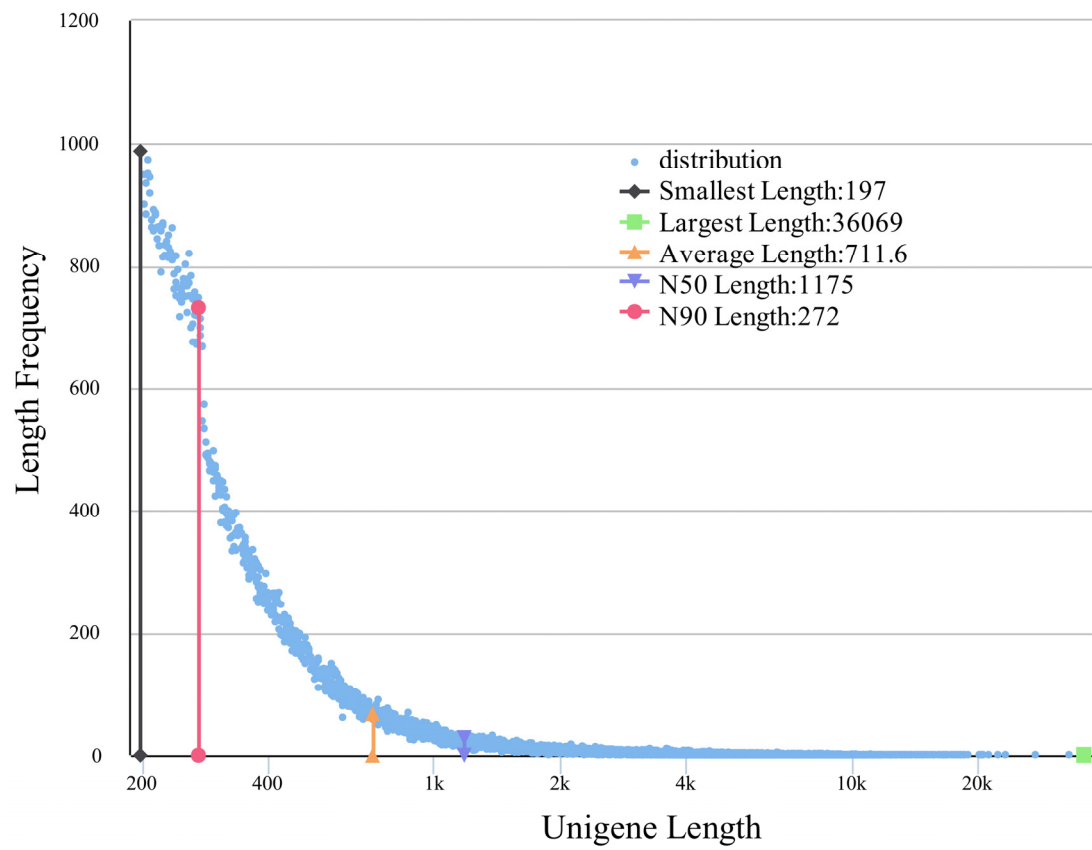

Figure S2. Length distribution of *P. clarkii* hepatopancreas unigenes.

Supplement: Supplementary file 1 [file antioxidants-11-01944-s001.zip › Figure S2.pdf]

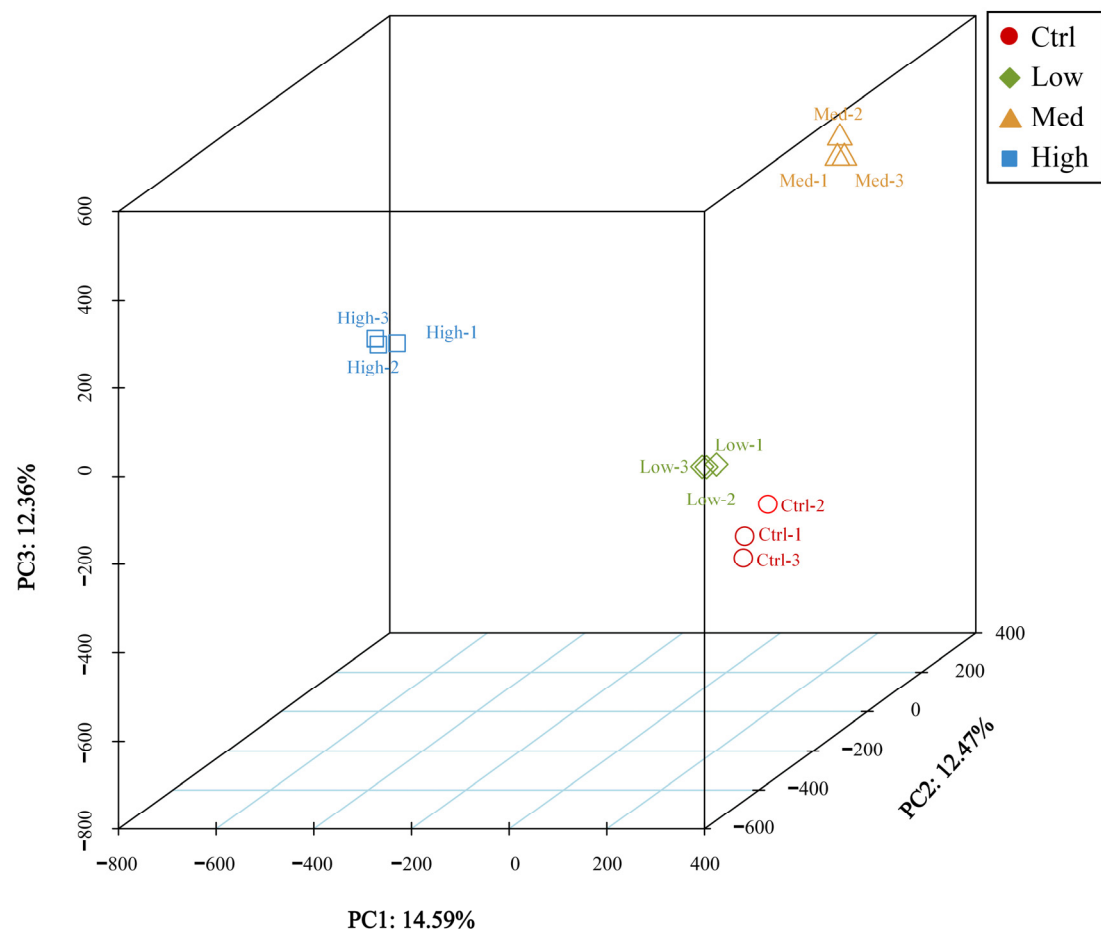

Figure S4. Principal Component Analysis (PCA) of the gene expression of samples.

Supplement: Supplementary file 1 [file antioxidants-11-01944-s001.zip › Figure S4.pdf]
